# Carbon footprint of clinical research: a systematic review and quantitative synthesis of estimates, sources of variation and emission hotspots

**DOI:** 10.1136/bmjopen-2026-122205

**Published:** 2026-10-05

**Authors:** Mitra Nekouei Shahraki, Lisa Van Bodegraven, Florine De Haes, Anneke Kwee, Nicolaas H Sperna Weiland, Wouter J K Hehenkamp, Mariska M Leeflang, Dorien Salentijn

**Affiliations:** 1 Department of Epidemiology and Data Science, Amsterdam University Medical Centres, Amsterdam, Netherlands; 2 Center for Sustainable Healthcare, Amsterdam University Medical Centres, Amsterdam, Netherlands; 3 Department of Fertility, Gynecology, and Obstetrics, OLVG, Amsterdam, Netherlands; 4 Program Health Care Evaluation and Appropriate Care, Zorginstituut Nederland, Diemen, Netherlands; 5 Department of Anesthesiology, Amsterdam University Medical Centres, Amsterdam, Netherlands; 6 Department of Obstetrics and Gynecology, Amsterdam University Medical Centres, Amsterdam, Netherlands; 7 Department of Radiology and Nuclear Medicine, Amsterdam University Medical Centres, Amsterdam, Netherlands

**Keywords:** EPIDEMIOLOGY, Methods, Systematic Review, Clinical Trial, PUBLIC HEALTH

## Abstract

**Abstract:**

**Objectives:**

To synthesise evidence on carbon dioxide equivalent (CO_₂_-eq) emissions associated with conducting clinical research, appraise its quality, explore variation and identify emission hotspots.

**Design:**

Systematic review and quantitative synthesis.

**Data sources:**

MEDLINE, Embase, Web of Science, HealthcareLCA, medRxiv and bioRxiv were searched from inception to November 2025 without language restrictions, with backward and forward citation searching.

**Eligibility criteria:**

Original studies estimating CO_₂_-eq emissions attributable to clinical research were eligible, regardless of study design or assessment method.

**Data extraction and synthesis:**

Two reviewers independently selected studies, extracted data on carbon-footprint assessments and the clinical studies they covered, and appraised the methodological and reporting quality of the assessments using a customized checklist. Emissions per clinical study were summarized descriptively by study and assessment characteristics and grouped into 10 activity categories to identify emission hotspots. Exploratory multivariable linear regression examined factors associated with log-transformed total CO₂-eq emissions per clinical study, accounting for clustering of clinical studies within assessments.

**Results:**

Ten carbon footprint assessments covering 45 clinical studies were included; six assessments were rated as high quality and four as medium quality. Emissions ranged from 8.8 to 3107 tCO₂-eq per clinical study, with a median of 78.0 tCO₂-eq (IQR 54.9-171.0). Descriptively, emissions per clinical study increased with the number of countries and sites and were higher when estimated using life cycle assessment (LCA) than non-LCA methods. In exploratory regression analyses, the number of countries, number of sites and assessment method accounted for the largest proportions of variation after adjustment for other factors (partial R²: 12.8%, 10.7% and 9.8%, respectively), although none of the associations was statistically significant. Meetings and travel, followed by clinical study unit operations, were the most frequent emission hotspots, although activity-level reporting was incomplete in several studies.

**Conclusions:**

Descriptively, carbon-footprint estimates increased with the geographical scale of clinical studies and varied across assessment methods, although no independent, statistically significant associations were established. Meetings and travel, as well as clinical study unit operations, were most frequently identified as emission hotspots, offering potential targets for mitigation, although hotspot identification was limited by incomplete activity-level reporting. As estimating emissions from clinical studies is increasingly encouraged, a standardized framework for prospective calculation could improve the reliability and comparability of estimates, facilitate hotspot identification, distinguish variation attributable to study characteristics from that arising from assessment methods, and support the systematic integration of carbon considerations and mitigation measures into study design and conduct.

**STRENGTHS AND LIMITATIONS OF THIS STUDY**

- A comprehensive search combined bibliographic databases, specialised and preprint sources and backward and forward citation tracking.
- Study selection and data extraction were performed independently by two reviewers, with methodological and reporting quality appraised using a tailored checklist.
- Activity-specific emissions were harmonised into predefined groups to facilitate hotspot comparisons across clinical studies.
- The 45 clinical-study estimates were derived from only 10 carbon-footprint assessments and therefore could not be considered fully independent throughout the synthesis.
- Variation in carbon-accounting methods, system boundaries, activity classifications and reporting limited direct comparability across assessments.

## Introduction

 Greenhouse gases are driving climate change, with serious consequences for human health. Addressing this health threat^1^ requires action from all sectors, not least in scientific practices and research. Clinical research is essential for generating evidence to improve human health, including evidence on the diagnosis and prognosis of health conditions and the safety and efficacy of health interventions.^2 3^ However, conducting clinical research itself requires resources and activities that can generate considerable greenhouse gas emissions.^4–6^ These environmental impacts can arise throughout the research process, from study planning and set-up through conduct, analysis, dissemination and implementation. Considering clinical research as a process comprising interconnected stages and activities provides a basis for identifying where environmental impacts arise and where they might be reduced. Integrating environmental considerations across this process could support more sustainable clinical research while preserving its scientific and societal value.^7^

Carbon footprint, expressed in carbon dioxide equivalents (CO_₂_-eq), is a commonly used quantitative measure of greenhouse gas emissions. The different stages of the clinical research process involve activities that contribute to CO_₂_-eq emissions, including study-related use of facilities and energy, clinical and laboratory procedures and supplies, staff and participant travel and computational activities. The environmental impact associated with these activities becomes increasingly relevant,^8 9^ as both the number and scale of clinical studies continue to grow.^10^ In parallel, interventions and tools to reduce these impacts are emerging.^9 11^ Ethical frameworks are also evolving to extend responsibility beyond individual protection, rights and safety to include communities and the environment.^12 13^ These developments underscore the need for valid and comparable approaches to quantifying clinical research emissions to quantify their magnitude, enable comparisons across studies and inform decisions about reducing their environmental impact.

While frameworks and initiatives for environmental sustainability have become increasingly established in laboratory research,^14 15^ comparable guidance for clinical research remains limited.^16 17^ Efforts to estimate emissions from clinical research can be traced to the Sustainable Trials Study Group convened by the London School of Hygiene and Tropical Medicine nearly two decades ago.^2^ Quantification nevertheless remains an emerging area of research,^5^ despite growing interest among researchers, policymakers and regulatory actors.^18 19^ Recent studies have expanded this evidence base across different clinical studies and fields, but methodological approaches and reported carbon-footprint estimates vary substantially.^5 6 20 21^ The extent to which this variation reflects characteristics of the clinical studies or methodological differences remains unclear.

The aim of this systematic review was to provide a comprehensive overview of the existing evidence base and to synthesise quantitative estimates of CO_₂_-eq emissions associated with the conduct of clinical research. The objectives were (1) to appraise the quality of the available evidence on CO_₂_-eq emission estimates to inform future measurement practices; (2) to derive summary estimates of the carbon footprint of clinical research; (3) to explore sources of variation and identify study characteristics associated with higher emissions; and (4) to identify major emission sources and hotspots for potential mitigation. Together, these objectives aim to inform the design and conduct of lower-carbon clinical research.^22^

## Methods

### Protocol, search strategy and selection criteria

This systematic review followed a predefined protocol registered in PROSPERO (CRD420251000783). We included original studies that estimated the carbon footprint associated with the conduct of clinical research and expressed the estimate in CO_₂_-eq. For this review, clinical research was defined as health (care) research involving human participants that investigates health, disease or healthcare. All clinical study designs were eligible, including interventional and observational studies, regardless of scale, duration, funding source or country. Clinical research-related carbon footprint was defined as the estimated CO_₂_-eq attributable to activities associated with the conduct of the clinical study. These included, for example, study-specific participant visits, sample collection and laboratory procedures, staff travel, materials and interventions and study monitoring and administration. Any methodological approach to estimating the carbon footprint was eligible.

Exclusion criteria included publications that did not provide an original carbon-footprint assessment based on data from the conduct of a clinical study (eg, estimates derived solely from previously published carbon-footprint estimates), and publications focusing exclusively on research outside the defined scope of clinical research (eg, preclinical laboratory or animal research). Detailed eligibility criteria are provided in online supplemental methods S-1. A comprehensive search strategy was developed by an information specialist and executed across all databases from inception through November 2025, without language restrictions to maximise the identification of relevant studies. The main search covered MEDLINE, Embase and Web of Science (search strategy detailed in online supplemental methods S-2). Additional searches included the HealthcareLCA database for relevant records, and medRxiv and bioRxiv to identify relevant preprints. Records retrieved from these sources were imported into EndNote and deduplicated before screening. Reference lists and citation tracking of included studies were screened using backward and forward snowballing^23^ via Google Scholar.

### Patient and public involvement

None.

### Data selection and extraction

Two reviewers (LvB and MNS) independently screened all titles and abstracts and subsequently assessed potentially eligible full-text reports. Disagreements regarding inclusion or exclusion at either stage were resolved through discussion and consensus. Data extraction was conducted independently by the same reviewers. Discrepancies were resolved through discussion and consensus. For example, when multiple carbon-footprint assessments reported estimates for overlapping clinical studies, data for the overlapping studies were extracted from the carbon-footprint assessment covering the largest number of clinical studies to avoid duplication; data for non-overlapping clinical studies were retained from the respective assessments. The study selection process is shown in the Preferred Reporting Items for Systematic Reviews and Meta-Analyses (PRISMA) flow diagram (online supplemental figure S1), and reporting follows the PRISMA reporting checklist for the systematic review (online supplemental table S7).^24^ Data were extracted at two levels: the carbon-footprint assessment level and the clinical study level. At the carbon-footprint assessment level, extracted variables included authors, publication year, conducting organisation and country, number of clinical studies assessed, carbon-footprint estimation method, data collection approach (real-time, retrospective or prospective) and estimated total CO_₂_-eq emissions. Where available, reported breakdowns of emissions by activity were also extracted and used for hotspot analysis.

At the clinical study level, extracted characteristics included study name, design, sample size, number of sites and countries and study duration (time between start and end dates). Data extraction for clinical studies was primarily based on information reported in the carbon-footprint assessment, in line with what was accounted for in the carbon-footprint estimation. When information was not reported, additional details were retrieved from external sources, including publications describing the clinical study or clinical trial registries. Clinical studies were also classified by hospital resource demand (low, medium or high), based on the intensity of clinical care and infrastructure required for study conduct. Classification was performed independently by two reviewers, with disagreements resolved through discussion and consensus. Detailed criteria and examples are provided in online supplemental methods S-3.

Where available, CO_₂_-eq emissions reported by activity were extracted and classified into 10 groups adapted from Griffiths *et al*^4^ who developed an activity-based framework for carbon-footprint assessment across the clinical trial lifecycle.

### Quality appraisal of carbon-footprint assessments

We assessed the methodological and reporting quality of CO_₂_-eq emission quantification in each carbon-footprint assessment using a customised checklist. In the absence of an established appraisal tool for this purpose, the checklist was informed by guidance on sustainable care pathways^25^ international life cycle assessment (LCA) standards (ISO 14040/14044),^26^ and developed iteratively through consensus discussions between an environmental and LCA expert (FdH) and a clinical epidemiologist and methodologist (MNS). The resulting checklist comprised eight domains reflecting key aspects of CO_₂_-eq quantification and reporting: (1) goal and scope clearly defined; (2) scope coverage (completeness); (3) activity data collection: transparent and reproducible; (4) activity data collection: real time; (5) using consistent standard emission factors; (6) assumptions clearly stated; (7) sensitivity or uncertainty analysis (accuracy); and (8) clear interpretation of findings and limitations. Further details on the development and application of the checklist are provided in online supplemental methods S-4, and the domain definitions and scoring criteria are presented in online supplemental table S1.

Each domain was scored independently by two reviewers (MNS and FdH) on a 3-point scale (2 points if fully met, 1 point if partially met and 0 points if not met), with discrepancies resolved through discussion and consensus. In the absence of an empirical basis for differential weighting, domains were equally weighted and summed to produce a summary score ranging from 0 to 16. The summary score was categorised using predefined thresholds as high (12–16), medium (8–11) or low (0–7) quality.

### Statistical analysis

#### Statistical synthesis (descriptive and stratified)

A descriptive synthesis of total CO_₂_-eq emissions across the 45 clinical studies was performed. Distributions were summarised using means and SDs and medians and IQRs. For selected categorical study and assessment characteristics, estimates were stratified by their predefined categories and summary measures were calculated within each stratum. These included number of countries, number of study sites, carbon-footprint assessment method (LCA or non-LCA), study design and quality appraisal category.

For pharmaceutical trials in which drug-production-related emissions were included in the carbon-footprint assessment scope and separately quantifiable, summary measures were calculated both with and without these emissions.

### Hotspots analysis

We performed a hotspot analysis to identify the study-related activity groups contributing the most to the total carbon footprint of each clinical study. In line with the data extraction approach, activity-specific tCO_₂_-eq estimates were harmonised into 10 predefined activity groups adapted from the framework proposed by Griffiths *et al*:^4,4^ trial set-up, clinical trial unit emissions, trial-specific meetings and travel, treatment (intervention), data collection and exchange, trial supplies and equipment, trial-specific patient assessments, samples, laboratory and trial close out. Where clinical studies used different activity classifications, reported activities were mapped to the most appropriate predefined group based on their descriptions. Where emissions for an activity group were not reported, they were coded as N/A and were not assumed to represent zero emissions. For each clinical study, the activity group with the highest reported emission estimate was classified as the emission hotspot. The number of times each activity group was identified as the emission hotspot across the 45 clinical studies was then calculated.

### Multivariable linear regression

Associations between clinical study characteristics and total carbon footprint across 45 clinical studies were examined using multivariable linear regression. Variables included sample size, number of countries, number of sites, study duration, hospital resource demand and carbon-footprint assessment method. Due to right-skewness of total CO_₂_-eq emissions, the outcome was log-transformed prior to analysis. To account for clustering of clinical studies within carbon-footprint assessments, CIs and p values were estimated using CR2 cluster-robust variance estimation with Satterthwaite small-sample correction.^27 28^ Model performance was summarised using adjusted R² and partial R² for individual variables. Multicollinearity among variables included in the fitted model was assessed using generalised variance inflation factors (GVIFs).^29^ Given the limited number of primary studies and assessment clusters, regression analyses were considered exploratory. All analyses were conducted in RStudio (V.2025.05).

## Results

### Search results and study characteristics

We included 10 carbon-footprint assessments^4 30–38^ reporting on a total of 45 clinical studies, as summarised in online supplemental table S2. Most studies were conducted by academic or public institutions in high-income countries, primarily the UK and Europe, with a smaller number from low- and middle-income settings (including Mali, South Africa and India). Industry-sponsored studies, mainly by Janssen and AstraZeneca, were typically larger, multicentre and multinational, whereas academic studies were more often single-country.

Carbon footprint assessments were published between 2009 and 2025, predominantly after 2020, and were conducted either by the clinical studies themselves or by independent academic groups; industry-sponsored trials were more often assessed internally. Most clinical studies were randomised controlled trials, with some pragmatic designs. Pragmatic trials evaluate interventions under routine, real-world clinical conditions. Assessments were mainly based on retrospective data, with some prospective elements such as real-time tracking of travel or energy use. Industry-led studies predominantly used LCA, while academic studies more often applied emission-factor-based approaches or activity-based audits.

Sample sizes of clinical studies ranged from 39 to 20 211 participants. Hospital resource demand was classified as low in 23 clinical studies, medium in 13 clinical studies and high in 9 clinical studies. Estimated total trial-related emissions ranged from 8.8 to 3107 tCO_₂_-equivalent per trial and from 0.01 to 9.17 tCO_₂_-equivalent per participant, with higher per-participant emissions observed in smaller, more resource-intensive trials.

### Quality appraisal of carbon-footprint assessments

Table 1 summarises the quality-appraisal results for the 10 included carbon-footprint assessments. Total scores ranged from 8 to 15 out of a maximum of 16, with all assessments classified as medium or high quality. Six carbon-footprint assessments were classified as high quality and four as medium quality, with higher scores generally observed in more recent assessments^36 38^ and lower scores in earlier publications.^33 37^ Most assessments clearly defined their goal and scope and demonstrated comprehensive scope coverage. Transparency and reproducibility of activity-data collection were generally adequate, although two assessments only partially met this criterion. In contrast, greater variability was observed in the use of consistent standard emission factors and the explicit reporting of assumptions.^4^ Sensitivity or uncertainty analyses were not consistently conducted, including in both Griffiths *et al* assessments.^4 34^ The use of real-time activity data were limited across all assessments.

**Table 1:** Methodological and reporting quality appraisal of included carbon-footprint assessments

Carbon-footprint assessment (author); clinical studies included; total quality score (rating)	Goal and scope clearly defined	Scope coverage (completeness)	Activity data collection: transparent and reproducible	Activity data collection: real time	Using consistent standard emission factors	Assumptions clearly stated	Sensitivity or uncertainty analysis (accuracy)	Clear interpretation of findings and limitations
LaRoche *et al*^32^ 7 clinical studies, 14 (high)	2	2	2	1	1	2	2	2
Smit *et al*^36^ 1 clinical study, 15 (high)	2	2	2	1	2	2	2	2
Cranley *et al*^30^ 6 clinical studies, 14 (high)	2	2	2	1	1	2	2	2
Siegfried *et al*^35^ 1 clinical study, 12 (high)	2	2	2	1	1	1	1	2
Griffiths *et al*^4^ 10 clinical studies, 9 (medium)	2	2	1	1	1	1	0	1
Griffiths *et al*^4^ 2 clinical studies, 12 (high)	2	2	2	1	1	2	0	2
Fougerou-Leurent *et al*^31^ 1 clinical study, 12 (high)	2	1	0	1	2	2	2	2
Mackillop *et al*^34^ 3 clinical studies, 11 (medium)	2	2	2	1	1	1	0	2
Subaiya *et al*^37^ 2 clinical studies, 8 (medium)	2	1	1	1	1	1	0	1
Lyle *et al*^33^ 12 clinical studies, 10 (medium)	1	1	1	1	1	2	2	2

The methodological and reporting quality of CO_₂_-eq quantification in the included carbon-footprint assessments was evaluated across eight domains: (1) clarity of goal and scope definition, including the unit of analysis and study boundaries; (2) completeness of scope coverage across relevant clinical-study-related activities; (3) transparency and reproducibility of activity-data collection; (4) use of real-time activity data; (5) consistency and reliability of emission-factor sources; (6) clarity and justification of methodological assumptions; (7) consideration of sensitivity or uncertainty related to assumptions or missing data; and (8) clarity of results interpretation and reporting of limitations. Each domain was scored as 2 (fully met), 1 (partially met) or 0 (not met or unclear). Total scores ranged from 0 to 16 and were classified as high (12–16), medium (8–11) or low (0–7) quality.

CO_2_-eq, carbon dioxide equivalents.

### Statistical synthesis (descriptive and stratified)

Table 2 presents overall and stratified descriptive summaries of carbon-footprint estimates across 45 clinical studies. Overall, the mean carbon footprint was 341.5 tCO_₂_-eq (SD 658.8), with a substantially lower median of 78.0 tCO_₂_-eq (IQR 54.9–171.0), reflecting the right-skewed distribution of estimates.

**Table 2:** Descriptive synthesis of carbon-footprint estimates across clinical studies, overall and stratified by clinical-study and assessment characteristics

Aggregated summary (N primary studies)		Summary	Tonnes CO_₂_-eq
All (N=45)		Mean (SD) Median (IQR)	341.5 (658.8) 78 (54.9–171)
Stratified summary			
Number of countries	1 (N=27)	Mean (SD) Median (IQR)	61.1 (38.4) 54.9 (26.3–86.2)
	2–5 (N=8)	Mean (SD) Median (IQR)	95.2 (53.1) 90 (66.5–108.0)
	6–10 (N=3)	Mean (SD) Median (IQR)	568 (203) 580 (359–765)
	>10 (N=7)	Mean (SD) Median (IQR)	1608 (909) 1437 (925–2498)
Number of sites	1 (N=16)	Mean (SD) Median (IQR)	66.9 (31.0) 73.4 (44.4–88.7)
	2–25 (N=11)	Mean (SD) Median (IQR)	64.0 (65.1) 37.8 (25–72)
	25–125 (N=11)	Mean (SD) Median (IQR)	213 (247) 106 (61–359)
	>125 (N=7)	Mean (SD) Median (IQR)	1608 (909) 1437 (925–2498)
Carbon-footprint assessment method	LCA (N=12)	Mean (SD) Median (IQR)	934.5 (1045.1) 469.5 (136.2–1487.3)
	Non-LCA (N=33)	Mean (SD) Median (IQR)	126.1 (204.0) 72.9 (46.7–101.5)
Study design	RCT (N=30)	Mean (SD) Median (IQR)	475 (777) 98.7 (27.6–562)
	Pragmatic trial (N=15)	Mean (SD) Median (IQR)	75.5 (21.1) 72.9 (59.5–88.7)
Quality assessment results	High (N=18)	Mean (SD) Median (IQR)	339.1 (746.3) 63.5 (27.6–198.8)
	Medium (N=27)	Mean (SD) Median (IQR)	343.4 (608.5) 86.2 (59.5–110.9)
	Low (N=0)	Mean (SD) Median (IQR)	–
Drug-production-related emissions	Reported total (N=11)	Mean (SD) Median (IQR)	1016.6 (1,054.8), 580.0 (189.5–1537.5)
	Excluding drug-production-related emissions (N=11)	Mean (SD) Median (IQR)	728.8 (752.5) 347.3 (121.3–402.3)

Values are reported in tonnes of CO_₂_-equivalent (CO_₂_-eq) emissions as mean (SD) and median (IQR). Stratified summaries are presented by number of countries, number of study sites, study design, quality appraisal category and inclusion of drug-production-related emissions. The latter was restricted to pharmaceutical trials for which drug-production-related emissions were included in the carbon-footprint assessment scope and separately quantifiable (N=11); estimates excluding these emissions were calculated by subtracting the drug-production-related component from the reported total CO_₂_-eq estimate. All summaries are descriptive and based on the clinical study estimates.

CO_₂_-eq, carbon dioxide equivalent; LCA, life cycle assessment; N, number of clinical studies; RCT, randomised controlled trial.

With increasing numbers of participating countries and study sites, carbon-footprint estimates progressively increased, reflecting greater geographical reach and a larger number of study locations, respectively. Median emissions increased from 54.9 tCO_₂_-eq in single-country studies to 1437 tCO_₂_-eq in studies conducted in more than 10 countries, and from 73.4 tCO_₂_-eq in single-site studies to 1437 tCO_₂_-eq in studies with more than 125 sites. Median emissions were higher in LCA-based than non-LCA assessments (469.5 vs 72.9 tCO_₂_-eq) and in randomised controlled trials (RCTs) than pragmatic trials (98.7 vs 72.9 tCO_₂_-eq). No clear descriptive pattern in carbon-footprint estimates was observed across quality appraisal categories.

The inclusion and reporting of drug-production-related emissions varied across clinical studies: these emissions were separately quantifiable in 11 studies, while in the remaining studies they were either excluded from the assessment scope, not separately reported, or not applicable (online supplemental table S3). Among the 11 pharmaceutical trials with separately quantifiable emissions, their exclusion reduced the median carbon-footprint estimate from 580.0 to 347.3 tCO_₂_-eq, corresponding to a 40.1% reduction (study-level estimates are presented in online supplemental table S4).

### Hotspots analysis

Online supplemental table S6 summarises the distribution of CO_₂_-eq emissions across predefined activity groups for the 45 clinical studies. A hotspot was assigned to each study based on the highest-emitting activity among those reported. Meetings and travel (the highest-emitting activity in 17 studies) and clinical study unit operations (the highest-emitting activity in 13 studies) most frequently emerged as emission hotspots. These patterns were most commonly observed in studies involving a larger number of sites and/or multiple countries. However, activity-level reporting was incomplete in several studies, particularly those using non-LCA approaches. Unreported activity groups were coded as not available rather than as zero; consequently, for these studies, the assigned hotspot represents the highest-emitting activity among those reported and may have been influenced by incomplete disaggregation.

Less frequently, treatment-related activities and study-specific patient assessments were identified as emission hotspots in four and three studies, respectively. These hotspots occurred in studies with higher hospital resource demand, involving more intensive clinical procedures, inpatient care or frequent follow-up. Studies classified as having low hospital resource demand, particularly outpatient or community-based trials, more often had emissions dominated by meetings and travel or clinical study unit operations.

### Multivariable linear regression

As shown in table 3, no statistically significant associations were observed between the examined variables and carbon-footprint per clinical study. CIs, which accounted for clustering of clinical studies within carbon-footprint assessments and incorporated a small-sample correction, were generally wide, indicating considerable uncertainty around the estimates. P values did not indicate any statistically significant associations. There was no evidence of substantial multicollinearity among the variables included in the model (adjusted GVIFs 1.28–2.25; online supplemental table S5). The estimates for the number of participating countries and study sites were both positive, and these variables had the largest partial R² values (12.8% and 10.7%, respectively), indicating that they accounted for the largest additional proportions of variation in the outcome conditional on the other variables in the model. The carbon-footprint assessment method had the next largest partial R² (9.8%), with the estimate indicating higher emissions for LCA-based than non-LCA assessments. The adjusted R² of the underlying linear regression model was 0.65, suggesting that the included variables collectively accounted for approximately 65% of the observed variation in log-transformed tCO_₂_-eq emissions.

**Table 3:** Multivariable linear regression of associations between clinical study characteristics and estimated carbon footprint (tCO_₂_-eq emissions), accounting for clustering of clinical studies within carbon-footprint assessments

Variable		β coefficient	CR2-adjusted 95% CI	CR2-adjusted p value	Partial R² (%)
Intercept		3.35	(1.72 to 4.99)	0.00	–
Sample size (per 10 persons)		−0.00	(−0.00 to 0.00)	0.79	0.5
Countries		0.06	(−0.10 to 0.23)	0.29	12.8
Sites		0.005	(−0.01 to 0.02)	0.34	10.7
Duration (years)		0.10	(−0.19 to 0.40)	0.34	5.5
Hospital resource demand	High	−0.44	(−4.03 to 3.15)	0.69	5.6
	Medium	0.20	(−1.47 to 1.87)	0.73	
	Low: reference	–	–	–	
Carbon-footprint assessment method	LCA	0.91	(−0.80 to 2.62)	0.19	9.8
	Non-LCA: reference	–	–	–	

β coefficients represent estimated associations with log-transformed total CO_₂_-eq emissions. Continuous variables were modelled per unit increase, with sample size expressed per 10 participants; categorical variables were compared with the indicated reference categories. CIs and p values account for clustering of clinical studies within carbon-footprint assessments using CR2 cluster-robust variance estimation with Satterthwaite small-sample correction. The adjusted R² of the underlying linear regression model was 0.65. Partial R² values were calculated from the underlying linear regression model as descriptive measures of the additional variation associated with each variable, conditional on the other variables in the model. Given the limited number of primary studies and assessment clusters relative to the number of variables, the multivariable analysis was considered exploratory and results should be interpreted cautiously.

CO_₂_-eq, carbon dioxide equivalent; CR2, bias-reduced cluster-robust variance estimator; LCA, life cycle assessment; R², coefficient of determination; t, tonnes.

## Discussion

In this systematic review, we identified 10 carbon-footprint assessments covering 45 clinical studies and found substantial variation in the emissions associated with conducting clinical research. The available assessments were of medium or high quality, although the reporting of assumptions and the use of sensitivity or uncertainty analyses remained inconsistent, and real-time activity data were rarely used. Across clinical studies, estimates ranged from 8.8 to 3107 tCO_₂_-eq per study, with a median of 78.0 tCO_₂_-eq (IQR 54.9–171.0) and varied considerably according to study characteristics and carbon-assessment methods. Studies involving more countries and study sites generally had higher emissions, while LCA-based assessments reported higher estimates than non-LCA assessments. In the multivariable regression analysis, these three factors accounted for the largest additional proportions of variation, but none of the examined associations was statistically significant. Meetings and travel together with clinical study unit operations most frequently emerged as emission hotspots, particularly among multinational and multisite studies and may offer potential targets for designing and conducting lower-carbon clinical research. Overall, the evidence base remains limited and methodologically inconsistent, highlighting the need for a standardised and transparent framework for assessing the carbon footprint of clinical research.

The findings from the descriptive summaries measures and multivariable analyses for the number of participating countries and clinical sites suggest that the geographical scale of clinical studies may contribute to variation in their carbon footprint, although neither association was statistically significant. The limited df available in our data further limit our ability to draw conclusions. However, the observed patterns could be explained by the additional coordination, parallel infrastructure, logistics, monitoring and travel that expansion across countries and sites may require.^9 32 37^ As multisite expansion is often motivated by recruitment challenges,^39^ strengthening local research infrastructure and improving recruitment and retention may reduce the need for broad geographical expansion. However, decisions about the number and location of sites must also consider recruitment feasibility, the demographic diversity and representativeness of the study population^40^ and equitable geographical access to clinical trial participation for eligible patient populations^41^; efforts to reduce emissions should therefore be pursued without compromising these scientific and ethical requirements.

The observed variation in carbon-footprint estimates may reflect, on the one hand, differences in clinical study characteristics and conduct and, on the other, methodological differences in carbon-footprint assessment. These sources of variation could not be fully disentangled in the available evidence. Descriptive summary measures were higher in LCA-based than in non-LCA assessments, and the use of an LCA-based assessment accounted for the next-largest additional proportion of variation in the multivariable model. This difference could reflect either the broader system boundaries and more comprehensive coverage of emission sources commonly used in LCA-based assessments or genuinely greater carbon intensity among the clinical studies assessed using these methods. LCA-based assessments were also used more often for larger industry-led studies, making it difficult to separate the influence of the assessment method from that of the studies being assessed. Beyond their classification as LCA-based or non-LCA-based, assessments differed in their system boundaries, included emission domains, activity groupings, assumptions, emission factors and reporting formats. Although all assessments were classified as medium or high quality, explicit reporting of assumptions and the application of sensitivity or uncertainty analyses were inconsistent. Moreover, most assessments relied largely on retrospective information, and real-time activity data collection was uncommon.

Similarly, descriptive summary estimates were higher for RCTs than for pragmatic trials; however, the available evidence was too limited to draw conclusions about an independent effect of study design. Within this limited evidence base, study design also overlapped with sponsor type, trial phase, geographical scale and the inclusion of intervention-manufacturing emissions, making these influences difficult to separate. Pragmatic trials may rely more extensively on existing care infrastructure, whereas RCTs can require study-specific procedures, monitoring and data collection.^42^ In our review, many RCTs were early-phase pharmaceutical trials that included drug manufacturing emissions, further increasing attributable impact and demonstrates the importance of assessment scope. Drug-production-related emissions were separately quantifiable in only 11 pharmaceutical trials, and their exclusion reduced the median carbon-footprint estimate in these trials by 40.1%. Intervention manufacturing may include active pharmaceutical ingredient production, formulation, packaging, labelling and placebo production. Whether and to what extent these emissions should be attributed to a clinical study depends partly on the relationship between manufacturing and study conduct. Their inclusion may be more readily justified when the intervention or placebo is manufactured specifically for the study, in early-phase clinical trials or irrespective of trial phase, whereas attribution is less straightforward when products are obtained from an existing supply or when production is shared with routine clinical use. Including manufacturing may substantially broaden the assessment scope and increase attributable emissions, a consideration that may also extend to medical devices and other health technologies. Transparent justification of system boundaries is therefore essential, particularly when emission sources are excluded, shared with routine care or only partly attributable to the research. Similar questions of assessment scope may apply to other activities. Food-related and nutrition-related emissions were not considered in the carbon-footprint assessments included in this review or in the activity framework proposed by Griffiths *et al*.^4^ However, previous studies have considered these emissions in carbon-footprint assessments of care pathways. Future research should explore whether and under what circumstances they should be included within the scope of carbon-footprint assessments of clinical research.

Meetings and travel most frequently emerged as the dominant emission hotspot. This category included site visits, monitoring, audits, investigator meetings, conferences and their associated logistics. Air travel can contribute disproportionately to travel-related emissions compared with other transport modes,^43 44^ indicating opportunities to reduce emissions by limiting unnecessary flights. Strategies such as prioritising virtual or hybrid formats, reducing non-essential travel, expanding remote monitoring and shifting towards lower-carbon transport modes (eg, from air to rail) may substantially reduce emissions from this hotspot. Encouraging these changes (eg, rail travel) may require institutional incentives, flexible travel policies, suitable digital infrastructure and research timelines that allow lower-carbon travel options. Nevertheless, travel cannot always be eliminated, particularly where on-site training, quality assurance or collaboration with settings having limited digital infrastructure is necessary. Mitigation strategies should therefore distinguish essential travel from activities that can be replaced or reorganised without affecting study quality or equitable collaboration.

Clinical study unit operations constituted the other most frequently identified hotspot, reflecting emissions associated with coordination, data management, office activities and prolonged staff engagement. Improving energy efficiency, using lower-carbon digital infrastructure and limiting non-essential data collection may help reduce these emissions. Excessive data collection increases the demands associated with data capture, monitoring, storage and processing without necessarily improving scientific value. Overall, aligning data collection with prespecified study objectives and adopting risk-based monitoring approaches could therefore improve methodological efficiency while reducing the associated environmental impacts. In studies with greater hospital resource demand, patient assessments and intervention-related activities more often emerged as important hotspots. This suggests that mitigation priorities may differ according to the clinical context: logistical and coordination activities may be more relevant in geographically dispersed, lower-resource-demand studies, whereas clinical procedures, inpatient care and intervention delivery may require greater attention in resource-intensive studies. Logistical and operational choices, reflecting organisational structures and behavioural practices, represent important targets for reducing emissions. System-level and behavioural changes may therefore complement technical and infrastructural interventions.^45 46^ These findings, however, are based predominantly on evidence from high-income settings. Emission profiles and feasible mitigation strategies may differ in low- and middle-income settings because of differences in research infrastructure, energy systems, travel requirements, decentralised care models, regulatory processes and capacity constraints. Mitigation approaches developed in high-income settings should therefore not be assumed to be directly transferable and should be adapted without shifting burdens to participating sites or undermining capacity strengthening and equitable research participation.

A standardised and transparent framework could provide guidance on defining system boundaries, selecting and grouping emission sources, choosing emission factors, attributing shared activities, handling assumptions and missing data, conducting uncertainty analyses and reporting estimates. Existing initiatives, such as the UK Greener Trials group, may offer a useful basis for further work and collaboration in this area.^44^ Such a framework should be sufficiently consistent to improve comparability while remaining adaptable to differences in study design, intervention type, clinical field and setting. Prospective assessment at the protocol-development stage could help identify anticipated hotspots and support targeted mitigation before implementation. Real-time monitoring during study conduct could subsequently determine whether planned reductions are achieved and allow mitigation strategies to be adapted when necessary. These developments would move carbon assessment beyond retrospective accounting towards its use as a practical tool for lower-carbon study design. However, the broader implications for research governance, reporting standards, training, legislation and regulation extend beyond the evidence directly examined in this review and should be regarded as areas for further methodological development and empirical evaluation. Within this broader agenda, carbon accountability could also extend to research dissemination, as emissions associated with unpublished studies may represent avoidable environmental waste. Clinical researchers may have an important role through decisions made during study design and conduct, but responsibility would also extend to methodologists, institutions, regulators and guideline developers. Future work should examine how environmental considerations can be incorporated into these structures without compromising scientific validity, participant safety or equity.

### Strengths and limitations

A strength of this review was the development and application of a tailored quality-appraisal tool, allowing emission estimates to be synthesised and compared across their methodological quality. We also harmonised reported emission sources into common categories, enabling the identification of recurrent hotspots and potential targets for mitigation across clinical studies. By combining quality appraisal, quantitative synthesis, exploratory multivariable analysis and hotspot assessment, this review places carbon-footprint estimates for clinical research within their methodological and operational context, showing not only their magnitude but also how they vary, where emissions arise, and where the evidence needs strengthening.

Many limitations should also be considered. The tailored quality-appraisal checklist and its classification thresholds were not formally piloted or externally validated. Although assessments were appraised independently by two reviewers and discrepancies were resolved through consensus, inter-rater agreement was not quantified. Formal evaluation of the checklist’s validity and reliability would therefore be a valuable area for future research. Although the synthesis included emission estimates for 45 clinical studies, these were derived from only 10 published carbon-footprint assessments. Estimates originating from the same assessment may share methodological choices, assumptions and emission factors and therefore cannot be considered fully independent. Although we accounted for this dependence in the multivariable analyses using cluster-adjusted CIs and p values, the small number of assessment-level clusters still limited statistical precision. Moreover, this dependence could not be accounted for in all parts of the synthesis. The observed variation reflects not only differences in clinical study characteristics but also differences in assessment methods, system boundaries, included emission sources and other researcher-defined choices. Not all of these sources of variation could be measured or accounted for in the synthesis. Most assessed clinical studies were conducted in high-income settings. This may partly reflect the geographical distribution of clinical research; however, the transferability of the resulting estimates and identified hotspots to settings with different research infrastructure, energy systems, travel patterns and clinical practices remains uncertain. Finally, carbon-footprint assessments may be more likely to be undertaken and published by institutions and researchers with greater capacity or interest in carbon accounting, although its influence on the resulting carbon-footprint estimates is uncertain.

## Conclusion

The available evidence indicates that clinical research can generate considerable carbon emissions, with substantial variation across studies. Descriptively, estimates increased with the geographical scale of clinical studies and varied across carbon-footprint assessment methods, although no statistically significant independent associations were established in exploratory multivariable analyses. Meetings and travel, together with clinical study unit operations, were recurrent emission hotspots and potential targets for mitigation, but incomplete activity-level reporting constrained their identification. Potential mitigation measures require evaluation in specific research settings before broader recommendations for their implementation can be made. For pharmaceutical trials whose assessments included and separately reported drug-production emissions, removing these emissions substantially lowered carbon-footprint estimates, highlighting the importance of consistently defined and transparently reported assessment boundaries. The limited evidence base and methodological heterogeneity of assessments highlight the need for a standardised, transparent assessment framework for prospective estimation that remains adaptable across study designs and settings. As interest in estimating health and care research emissions grows, such a framework could strengthen the reliability and comparability of estimates, help distinguish variation related to study characteristics from that arising from assessment differences, and improve hotspot identification. This could support the integration of carbon considerations and mitigation measures into the design and conduct of health and care research.

## Supplementary material

10.1136/bmjopen-2026-122205 online supplemental file 1

## Data Availability

Data are available upon reasonable request.

## References

[1] World Health Organization (2021). Climate change and health: World Health Organization. https://www.who.int/news-room/fact-sheets/detail/climate-change-and-health.

[2] Sustainable Trials Study Group (2007). Towards sustainable clinical trials. BMJ.

[3] McMichael AJ, Bambrick HJ (2007). Greenhouse-gas costs of clinical trials. The Lancet.

[4] Griffiths J, Adshead F, Al-Shahi Salman R (2024). What is the carbon footprint of academic clinical trials? A study of hotspots in 10 trials. BMJ Open.

[5] You F, Coffey T, Powell D (2025). Carbon emissions associated with clinical trials: a scoping review. J Clin Epidemiol.

[6] Billiones R (2022). Carbon footprint of clinical trials: a high-level literature review. Medical Writing.

[7] World Health Organization (2026). Clinical trials and environmental sustainability: review of key considerations to develop climate change mitigation and adaptation strategies. https://iris.who.int/server/api/core/bitstreams/e676dd94-f47d-4285-885d-a039332e5f3d/content.

[8] Mittra S, Baraskar S, Wolff-Holz E (2024). Aligning environmental, social, and governance to clinical development: moving towards more sustainable clinical trials. *GaBI J*.

[9] Tao Y, Steckel D, Klemeš JJ (2021). Trend towards virtual and hybrid conferences may be an effective climate change mitigation strategy. Nat Commun.

[10] ClinicalTrials.gov Trends and charts on registered studies. https://clinicaltrials.gov/about-site/trends-charts.

[11] Pickles K, Haas R, Guppy M (2024). Clinician and health service interventions to reduce the greenhouse gas emissions generated by healthcare: a systematic review. BMJ Evid Based Med.

[12] Ross LF, Loup A, Nelson RM (2010). Human Subjects Protections in Community-Engaged Research: A Research Ethics Framework. Journal of Empirical Research on Human Research Ethics.

[13] Samuel G, Richie C (2023). Reimagining research ethics to include environmental sustainability: a principled approach, including a case study of data-driven health research. J Med Ethics.

[14] Freese T, Elzinga N, Heinemann M (2024). The relevance of sustainable laboratory practices. *RSC Sustain*.

[15] Schell BR, Bruns N (2024). Lab sustainability programs LEAF and My Green Lab®: impact, user experience & suitability. *RSC Sustainability*.

[16] Borza VA, Chen Q, Clayton EW (2025). Computational strategic recruitment for representation and coverage studied in the All of Us Research Program. NPJ Digit Med.

[17] Lannelongue L, Aronson H-EG, Bateman A (2023). GREENER principles for environmentally sustainable computational science. *Nat Comput Sci*.

[18] Blom IM, Beagley J, Quintana AV (2022). The COP26 health commitments: A springboard towards environmentally sustainable and climate-resilient health care systems?. *The Journal of Climate Change and Health*.

[19] AstraZeneca (2021). Ambition zero carbon: Our path to net zero.

[20] Merson L, Witt K, Hussaini A (2024). Infrastructure, capabilities, and capacities required for clinical trials design and delivery: A rapid scoping review of recommendations and regulations. Wellcome Open Res.

[21] Jean C, Layese R, Canouï-Poitrine F Assessing the carbon footprint of clinical trials: a systematic review. medRxiv.

[22] De Soete W, Jiménez-González C, Dahlin P (2017). Challenges and recommendations for environmental sustainability assessments of pharmaceutical products in the healthcare sector. Green Chem.

[23] Wohlin C (2014). Guidelines for snowballing in systematic literature studies and a replication in software engineering.

[24] Page MJ, McKenzie JE, Bossuyt PM (2021). The PRISMA 2020 statement: an updated guideline for reporting systematic reviews. BMJ.

[25] Whiting A (2015). Care pathways: guidance on appraising sustainability.

[26] Standardization. IOf (2022). ISO 14040:2006 environmental management — life cycle assessment — principles and framework.

[27] McCaffrey DF, Bell RM (2003). Bias reduction in standard errors for linear regression with multi-stage samples. Quality control and applied statistics.

[28] Pustejovsky JE, Tipton E (2018). Small-Sample Methods for Cluster-Robust Variance Estimation and Hypothesis Testing in Fixed Effects Models. *Journal of Business & Economic Statistics*.

[29] O’brien RM (2007). A Caution Regarding Rules of Thumb for Variance Inflation Factors. *Qual Quant*.

[30] Cranley D, Dunn S, Taylor J-P (2025). Carbon footprint of a sample of clinical trials for people with neurological disorders: cross-sectional analysis. BMJ Open.

[31] Fougerou‐Leurent C, Forteau L, Perrot‐Loyer M (2026). Evaluating the Environmental Impact of Clinical Research: A Full Life Cycle Analysis of a French Academic Randomised Clinical Trial. Fundamemntal Clinical Pharma.

[32] LaRoche JK, Lanier J, Alvarenga R (2025). Climate footprint of industry-sponsored in-human clinical trials: life cycle assessments of clinical trials spanning multiple phases and disease areas. BMJ Open.

[33] Lyle K, Dent L, Bailey S (2009). Carbon cost of pragmatic randomised controlled trials: retrospective analysis of sample of trials. BMJ.

[34] Mackillop N, Shah J, Collins M (2023). Carbon footprint of industry-sponsored late-stage clinical trials. BMJ Open.

[35] Siegfried NL, Erasmus-Claassen L-A, Griffiths J (2025). CLIMATRIAL: applicability and adaptability of a novel method to quantify the carbon footprint of a South African clinical trial. Trials.

[36] Smit MJ, Mahamar A, Kooistra E (2025). Life cycle assessment of a clinical malaria trial in Mali reveals large environmental impacts of electricity consumption and international travel. *PLOS Sustain Transform*.

[37] Subaiya S, Hogg E, Roberts I (2011). Reducing the environmental impact of trials: a comparison of the carbon footprint of the CRASH-1 and CRASH-2 clinical trials. Trials.

[38] LaRoche JK, Alvarenga R, Collins M (2024). Climate footprint of industry-sponsored clinical research: an analysis of a phase-1 randomised clinical study and discussion of opportunities to reduce its impact. BMJ Open.

[39] Sertkaya A, Wong H-H, Jessup A (2016). Key cost drivers of pharmaceutical clinical trials in the United States. *Clin Trials*.

[40] Gross AS, Harry AC, Clifton CS (2022). Clinical trial diversity: An opportunity for improved insight into the determinants of variability in drug response. Br J Clin Pharmacol.

[41] Lee H, Bates AS, Callier S (2024). Analysis and Optimization of Equitable US Cancer Clinical Trial Center Access by Travel Time. JAMA Oncol.

[42] Roland M, Torgerson DJ (1998). Understanding controlled trials: What are pragmatic trials?. BMJ.

[43] Gössling S, Humpe A (2020). The global scale, distribution and growth of aviation: Implications for climate change. Glob Environ Change.

[44] Adshead F, Al-Shahi Salman R, Aumonier S (2021). A strategy to reduce the carbon footprint of clinical trials. The Lancet.

[45] Batcup C, Breth-Petersen M, Dakin T (2023). Behavioural change interventions encouraging clinicians to reduce carbon emissions in clinical activity: a systematic review. BMC Health Serv Res.

[46] Osbaldiston R, Schott JP (2012). Environmental sustainability and behavioral science: Meta-analysis of proenvironmental behavior experiments. Environ Behav.

