# Supplementary material for: Carbon footprint of clinical research: a systematic review and quantitative synthesis of estimates, sources of variation and emission hotspots
Source: BMJ Open. 2026 Oct 5;16(10):e122205. doi: 10.1136/bmjopen-2026-122205 (PMC13646982; doi:10.1136/bmjopen-2026-122205)
Supplement: Supplementary file 1 [file bmjopen-2026-122205.reviewer_comments.pdf]

PEER REVIEW HISTORY

BMJ Open publishes all reviews undertaken for accepted manuscripts. Reviewers are asked to complete a checklist review form and are provided with free text boxes to elaborate on their assessment. These free text comments are reproduced below.

ARTICLE DETAILS

Title (Provisional)

Carbon footprint of clinical research: a systematic review and quantitative synthesis of estimates, sources of variation and emission hotspots

Authors

Nekouei Shahraki, Mitra; van Bodegraven, Lisa; de Haes, Florine; Kwee, Anneke; Sperna Weiland, Nicolaas H; Hehenkamp, Wouter J.K.; Leeflang, Mariska; Salentijn, Dorien

---

VERSION 1 - REVIEW

**Reviewer**	**1**
**Name**	**Piffoux , Max**
**Affiliation**	**Hospices Civils de Lyon**
**Date**	**06-Jun-2026**
**COI**	**None**

I thank the authors for this very interesting study.

I have few comments that require revision or explanation:

1) Isn't there a significant correlation between number of centers, number of countries and number of patients ? doesn't it explain while the number of patient is not statistically significant ? Was that taken into account in the statisctical analysis (not described in the methods). I think this needs to be explored in details as it is the main result of the paper.

2) regarding : " In our review, several RCTs were early-phase pharmaceutical trials that included drug manufacturing emissions, further increasing attributable impact. These findings suggest that both study design and research context shape variation in carbon intensity." and "Whether intervention manufacturing should be included in all study-specific emission estimates remains conceptually debated; however, in early-phase contexts, production would not occur without the trial. This likely makes pharmaceutical trials broader in scope and potentially higher in attributable emissions, a consideration that may also extend to medical devices and other health technologies."

=> It seems this explains a lot of the carbon footprint in these studies and I do not clearly understand the argument regarding phase I units versus phase II-III trials as in each case manufacturing of the drug is needed, and would not be performed in the absence of clinical trial. Altogether, I suggest to perform a sensibility analysis excluding manufacturing emissions to see whether the main results are confirmed (maybe in sup data).

3) regarding "Although we synthesized evidence from 45 primary clinical studies, our data were drawn from 10 published studies, the majority conducted in high-income settings. Therefore, the representativeness of the findings for other contexts may be limited"

=> Most clincal studies are performed in rich countries, so I am not (fully) convinced by the argument.

Best regards

**Reviewer**	**2**
**Name**	**Arslan, Sedat**
**Affiliation**	**Bursa Uludag University**
**Date**	**07-Jun-2026**
**COI**	**None**

Dear Authors,

This manuscript addresses a timely and important issue: the environmental footprint of clinical research. The topic is highly relevant to BMJ Open because it connects research methodology, public health, sustainability, and clinical trial governance. The manuscript has several clear strengths, including a registered systematic review protocol, a broad search strategy, duplicate screening and extraction, a tailored quality appraisal approach, quantitative synthesis, regression modelling, and hotspot analysis. The finding that travel, meetings, study unit operations, international scope, and number of sites are major contributors to emissions is practically meaningful and could help trialists, funders, ethics committees, and research institutions integrate sustainability into clinical research planning.

However, I do not think the manuscript is ready for publication in its current form. The main concern is the strength of the quantitative conclusions relative to the small and heterogeneous evidence base. Although the authors analyse 45 primary clinical studies, these data come from only 10 published carbon-footprint assessment studies, and several observations are clustered within the same source study, institution, sponsor, or carbon-accounting framework. Treating the 45 primary studies as independent observations in a multivariable linear regression may overstate precision and increase the risk of spurious associations. The authors should either use a modelling strategy that accounts for clustering, such as a mixed-effects model or cluster-robust standard errors, or clearly downgrade the regression to an exploratory analysis. With several predictors and only 45 observations, the

model is also likely overfitted. This is particularly important because the manuscript presents R^2 and partial R^2 values in a way that may imply more certainty than the data can support.

A second major issue concerns the quality-weighted mean of 355.3 tCO_2-eq per clinical study. While this is an interesting descriptive estimate, its interpretation is problematic because the underlying studies differ greatly in design, scale, country coverage, trial phase, system boundaries, emission factors, and inclusion of intervention manufacturing. Weighting by a custom quality score may not solve this heterogeneity and may even create a false sense of methodological precision. I suggest presenting this estimate more cautiously, emphasizing the median and range, and using sensitivity analyses excluding extreme values, industry-sponsored trials, early-phase pharmaceutical trials, and studies with incomplete activity disaggregation.

The manuscript would also benefit from clearer distinction between variability caused by true operational differences and variability caused by carbon-accounting methodology. For example, higher emissions in LCA-based studies may reflect more complete capture of emission sources rather than genuinely more carbon-intensive research. This distinction should be made explicit throughout the results and discussion. Similarly, the finding that RCTs have higher emissions than pragmatic trials may be confounded by sponsor type, trial phase, intervention manufacturing, and international scale. The authors should avoid implying causality from these associations.

There appears to be an inconsistency in the reporting of the regression results for randomized controlled trials. The abstract reports $\beta=0.97$ with 95% CI 0.68–1.27, whereas the results/table appear to show $\beta=0.97$ with a confidence interval that is either incorrectly ordered or inconsistent. This must be corrected. All regression estimates, confidence intervals, p values, and interpretations should be checked carefully.

The quality appraisal tool is useful but requires further justification. Because it is customized and not externally validated, the authors should explain how the domains were selected, whether the scoring system was piloted, whether inter-rater agreement was assessed, and how subjective classifications such as hospital resource demand were handled. The high/medium/low hospital resource demand classification is potentially valuable, but the criteria need to be more transparent and reproducible. It would help to provide examples and explain how disagreements were resolved.

The hotspot analysis is one of the strongest and most practice-relevant parts of the paper, but it should be strengthened by explaining how activities were harmonized when studies used different categories. Some studies did not report disaggregated emissions, and this missingness could bias the hotspot findings. The authors should state whether hotspots were calculated only among studies with available disaggregation or whether missing categories were treated in another way.

The discussion is generally thoughtful, but it should be more balanced. The manuscript sometimes moves from descriptive evidence to broad recommendations for governance,

regulation, and research training without fully acknowledging the limited empirical base. These recommendations are reasonable, but they should be framed as implications requiring further development rather than direct conclusions from the current dataset. The authors should also expand the equity perspective. Most included evidence comes from high-income settings, and the carbon implications of research in low- and middle-income countries may differ substantially because of infrastructure, travel needs, decentralised care models, regulatory requirements, and capacity constraints.

The authors should consider citing additional recent literature on sustainability, public health, and implementation of sustainable health-related practices where appropriate. Relevant works that could strengthen the broader discussion include 10.62210/ClinSciNutr.2024.93, because it discusses the integration of sustainability-oriented approaches into clinical care and could help connect the proposed carbon-accounting framework with practical clinical implementation; 10.1007/s11356-023-27694-8, because it links food insecurity with sustainable and healthy behaviours and is relevant to the manuscript’s equity-oriented discussion of sustainability in health systems; 10.3389/fnut.2025.1684438, because it addresses knowledge-to-action pathways for sustainable health behaviours, which is relevant to translating carbon-footprint evidence into behavioural and institutional change; and 10.3389/fpubh.2025.1569755, because it highlights global vulnerability patterns across diet, health, and health-system indicators, which would support the authors’ acknowledgement that most current evidence comes from high-income settings. These citations should not be added superficially, but they are relevant if the authors expand the discussion around sustainability implementation, health equity, and public-health relevance.

The language is mostly understandable, but the manuscript requires careful editing for grammar, repetition, and precision. For example, there are repeated phrases in the methods, some awkward wording such as “this variation affect comparability,” and inconsistent use of “trial,” “clinical study,” and “clinical research.” The authors should standardize terminology and ensure that “clinical trial” is not used when the included evidence also covers broader clinical research.

Overall, this is a valuable and potentially publishable manuscript, but the current version overstates some quantitative conclusions and needs stronger methodological caution, clearer handling of clustering and heterogeneity, corrected regression reporting, improved explanation of the quality appraisal and resource-demand classification, and a more balanced discussion of generalizability.

Best regards

**Reviewer**	**3**
**Name**	**Doran, Peter**

Affiliation **University College Dublin School of Medicine and Medical Science, School of Medicine**

Date **19-Jun-2026**

COI **None**

This is a timely and relevant systematic review addressing an increasingly important topic: the environmental impact of clinical research. The manuscript is a valuable addition to the literature and has a number of strengths, including:

• A clearly defined research question.

• Systematic review methodology with PROSPERO registration.

• An attempt to move beyond narrative synthesis by incorporating quantitative analyses

• Practical implications for trial design and sustainability policy.

However , I would suggest the authors further consider some specific methodological and reporting issues.

1. Limited evidence base versus strength of conclusions

The review includes only 10 studies representing 45 primary clinical studies, and most originate from high-income countries and a relatively small number of research groups. The conclusions regarding average emissions, determinants of emissions, and hotspot patterns may therefore appear stronger. The authors should emphasise more strongly that the field remains highly immature. In particular the should frame the reported quality-weighted mean (355.3 tCO_2-eq) as a provisional estimate rather than a benchmark.

2. Quality appraisal tool requires stronger justification and validation

The review uses a customized 8-domain quality appraisal tool developed by the authors. While understandable and highly interesting given the lack of existing tools, the manuscript provides limited evidence that the instrument is reliable or validated. This generates some concerns including that the Domain weighting appears arbitrary, Equal weighting of all domains may not reflect their importance and the rationale for the cutoffs (high/medium/low quality) is unclear.

I would suggest that the authors expand the discussion to provide more detail on the development process of the tool, the pilot testing, inter-rater reliability measures and perhaps consider including sensitivity analyses showing if results change when quality weighting is omitted.

3. Statistical modelling

The multivariable regression includes multiple predictors despite habving just 45 observations which may introduce overfitting. The authors should report more details

including Variance inflation factors (VIFs), Residual plots and ensitivity analyses using simpler models.

4. Interpretation of causal relationships

There are a number of points at which discussion statements imply causal mechanisms – For example more countries → higher emissions…. more sites → higher emissions… RCTs → higher emissions

While these onservations are plausible and likely to be born out, the study design is observational and cross-sectional. The analyses demonstrate association rather than causation. I would encourage the authors to use more cautious language such as "associated with" , "may contribute to" and "consistent with the hypothesis that" rather than implying direct causality.

5. Heterogeneity in carbon accounting methods

The authors show a central finding is that methodological variation drives much of the observed variability. I think this could be explored in more detail, perhaps including a discussion of …

• How much variation is attributable to methodology versus trial characteristics?

• Are LCA-based studies systematically higher because they capture more sources?

Whilst these are in many ways complex questions, a really nice addition to the manuscript would be A table comparing carbon-accounting approaches with a sensitivity analysis stratified by accounting method.

6. hospital resource demand classification

The classification into low, medium, and high resource demand appears at least to my reading to be subjective. Can the authors provide explicit cirteria, with examples?

**Reviewer**	**4**
**Name**	**Gray, William**
**Affiliation**	**NHS Improvement, Getting It Right First Time**
**Date**	**25-Jun-2026**
**COI**	**None**

This is a well written study, and the findings are of interest. I have a few comments.

• I am confused by the description of 'study level' and 'primary clinical study level#. Could you say a bit more about this distinction. I assume it is to account for papers reporting multiple studies. Can I suggest picking one method and sticking to it to avoid confusion. I would have thought the primary clinical study level is the one to go for.

• The sentences below appear to broadly repeat the same information: “Primary clinical study characteristics include name, design, sample size, number of sites and countries, study duration (time between start and end dates). At the primary clinical study level, extracted characteristics included study name, design, sample size, number of sites and countries, and study duration (time between start and end dates).”

• The quality appraisal tool is novel and appears to have been well thought through and suitable for its primary purpose. See below, but I do, however, have concerns about its use as a weighting mechanism.

• Statistical methods: I am not convinced of the need for the linear regression analyses or the weighting for quality. Both methods are likely to have methodological flaws. For linear regression, it would be good to see evidence of validity assessment (eigenvalues and vector analysis, VIF calculation etc). I would have thought multicollinearity was an issue and it would be good to know if it was. Do the independent variables meet all the assumptions of linear regression? With regard to the weighting. How do you know that this crude weighting method is valid? I don’t think the scoring system, given that it is an ordinal scale, will have been designed to allow for weighting in this way. Should a study that scores 6 really have double the weighting of a study that scores 3? My feeling is that neither of these analyses add a huge amount and you could draw exactly the same conclusions with fewer caveats by simply reporting crude descriptive data. You don’t need a regression model to point out that an RCT has a higher per patient carbon footprint than a cohort study.

• Related to the above point, the authors report and discuss the quality weighted average (355 tonnes CO_2e, but place less emphasises on the crude average (mean or median), which I don’t think is mentioned to any great extent. This value is perhaps the more interesting one. In lieu of using the quality weighted average, you could report the average for studies meeting and not meeting a given quality score or graph how values of CO_2e vary across the quality scores. This may be of more value to readers and make a simpler and more digestible point.

• Was the outcome of interest total carbon emissions for the entire study, or per patient carbon emissions? Per patient makes more sense.

• The hotspot analysis adds value as it can lead to an informed discussion of mitigation, although the message seems to be that the hotspots are very study specific. It should perhaps be more of a focus.

• “Use of a life cycle assessment (LCA)-based carbon calculation system was marginally statistically significant and associated with higher estimated emissions compared with non-LCA approaches ($\beta = 0.97$; 95% CI: 0.00 to 1.96; $p = 0.05$).” This finding is difficult to interpret and raises the question as to whether it should be included in the model and the model design. The finding may just show that LCA methods estimate higher emissions or is it some (unaccounted for in modelling) characteristics of studies that used LCA. Most of the other

independent variables seem to be design properties of the study, but this is not and seems out of place.

• The discussion is well written. It has large sections on hotspot mitigation, which is good, but emphasises that this should be more of a focus of the results section.

• I agree on the need for a guiding framework on scope and boundaries for clinical studies. Such guidance is much needed to ensure consistence of reporting. The authors may wish to expand on this point. I know there are groups in the UK (e.g. Grener Trials) with an interest in this area. This point could also be linked to the paragraph immediately after on variability.

• One point the authors could pull out more in the discussion section early on is that the mean/median emissions of a trial are relatively high (355 tonnes CO2e). The authors may want to offer a comparison of what this means in terms of car journeys, hot meals or heating, just to place it into perspective. They could also compare it to routine clinical care, where some useful estimates exist.

• References 1, 7 and 27 appear wrongly and need to be corrected. I think you have used Endnote and not put a common after the entire organisation's name (e.g. World Health Organisation,)

VERSION 1 - AUTHOR RESPONSE

**Reviewer: 1**<br>Dr. Max Piffoux , Hospices Civils de Lyon<br>Comments to the Author:<br>I thank the authors for this very interesting study.<br>I have few comments that require revision or explanation<br>**Thank you very much, Dr Piffoux, for your thoughtful comments and assessment of our study. We have carefully addressed each comment and provided our point-by-point responses below.**	
1	Isn't there a significant correlation between number of centers, number of countries and number of patients ? doesn't it explain while the number of patient is not statistically significant ? Was that taken into account in the statisctical analysis (not described in the methods). I think this needs to be explored in details as it is the main result of the paper.<br>**Thank you very much for your comment.** We agree that the number of participants, countries, and study sites may be correlated. **In response to your comment, we assessed multicollinearity using generalized variance inflation factors (GVIFs). The adjusted GVIFs ranged from 1.28 to 2.25, providing no evidence of substantial multicollinearity.** We therefore found no indication that multicollinearity explained the nonsignificant estimate for sample size. We have now described the multicollinearity assessment in the Methods and reported the results in Table S-4. Separately, to account for the clustering of clinical studies within carbon-footprint assessments, we estimated confidence intervals and P values using CR2 cluster-robust variance estimation with a Satterthwaite small-

	sample correction. These revisions are reflected in the regression results presented in Table 4. Given the limited sample size and number of assessment-level clusters, we now describe the multivariable analysis as exploratory and interpret the findings cautiously.
2	2) regarding : " In our review, several RCTs were early-phase pharmaceutical trials that included drug manufacturing emissions, further increasing attributable impact. These findings suggest that both study design and research context shape variation in carbon intensity." and "Whether intervention manufacturing should be included in all study-specific emission estimates remains conceptually debated; however, in early-phase contexts, production would not occur without the trial. This likely makes pharmaceutical trials broader in scope and potentially higher in attributable emissions, a consideration that may also extend to medical devices and other health technologies."<br>=> It seems this explains a lot of the carbon footprint in these studies and I do not clearly understand the argument regarding phase I units versus phase II-III trials as in each case manufacturing of the drug is needed, and would not be performed in the absence of clinical trial. Altogether, I suggest to perform a sensibility analysis excluding manufacturing emissions to see whether the main results are confirmed (maybe in sup data).
3	3) regarding "Although we synthesized evidence from 45 primary clinical studies, our data were drawn from 10 published studies, the majority conducted in high-income settings. Therefore, the representativeness of the findings for other contexts may be limited"<br>=> Most clincal studies are performed in rich countries, so I am not (fully) convinced by the argument.<br><br>Thank you very much for your comments.<br><br>-We agree that study phase alone does not determine whether manufacturing emissions should be attributed to a clinical trial, as trial-specific drug manufacturing may also be required in phase II and III trials. The more relevant consideration is whether the intervention was manufactured specifically for the trial or obtained from an existing supply intended for routine use. **We have revised the Discussion accordingly and removed the implication that this issue is restricted to early-phase trials.**<br>*"Attribution may depend less on trial phase than on whether the intervention was manufactured specifically for the trial or obtained from an existing supply intended for routine care. Trial-specific manufacturing may occur across different phases and supports its inclusion within the study boundary, whereas attribution is less straightforward when products are shared with routine clinical use. Including manufacturing may substantially broaden the assessment scope and increase attributable emissions, a consideration that may also extend to medical devices and other health technologies"*<br><br>**In response to your suggestion, we examined the influence of drug-production emissions where these were separately quantifiable.** This was possible for 11 pharmaceutical trials. Excluding these emissions reduced the median carbon-footprint estimate from 580.0 to 347.3 tCO_2-eq, corresponding to a 40.1% reduction. We have added these results to the Results section and Table S-3. **A full sensitivity analysis of the multivariable model based on uniform exclusion of**

	**manufacturing emissions was not possible because these emissions were not separately identifiable across all 45 clinical studies.**<br><br>-We agree that the predominance of studies from high-income countries may partly reflect the geographical distribution of clinical research and does not necessarily indicate that the included evidence is unrepresentative of where most clinical studies are conducted. **We have therefore revised this limitation to focus more specifically on the transferability of the estimates and emission hotspots to settings with different infrastructure and practices.**<br><br>*"Most assessed clinical studies were conducted in high-income settings. This may partly reflect the geographical distribution of clinical research; however, the transferability of the resulting estimates and identified hotspots to settings with different research infrastructure, energy systems, travel patterns, and clinical practices remains uncertain"*

<table>
<tr><td colspan="2">

Reviewer: 2

Dr. Sedat Arslan, Bursa Uludag University
Comments to the Author:

Dear Authors,
This manuscript addresses a timely and important issue: the environmental footprint of clinical research. The topic is highly relevant to BMJ Open because it connects research methodology, public health, sustainability, and clinical trial governance. The manuscript has several clear strengths, including a registered systematic review protocol, a broad search strategy, duplicate screening and extraction, a tailored quality appraisal approach, quantitative synthesis, regression modelling, and hotspot analysis. The finding that travel, meetings, study unit operations, international scope, and number of sites are major contributors to emissions is practically meaningful and could help trialists, funders, ethics committees, and research institutions integrate sustainability into clinical research planning. However, I do not think the manuscript is ready for publication in its current form.
Thank you very much, Dr Arslan, for your thoughtful comments and for highlighting the relevance and strengths of our study. We have carefully considered each of your concerns and revised the manuscript accordingly. Our point-by-point responses are provided below.
</td></tr>
<tr><td>1</td><td>
The main concern is the strength of the quantitative conclusions relative to the small and heterogeneous evidence base. Although the authors analyse 45 primary clinical studies, these data come from only 10 published carbon-footprint assessment studies, and several observations are clustered within the same source study, institution, sponsor, or carbon-accounting framework. Treating the 45 primary studies as independent observations in a multivariable linear regression may overstate precision and increase the risk of spurious associations. The authors should either use a modelling strategy that accounts for clustering, such as a mixed-effects model or cluster-robust standard errors, or clearly downgrade the regression to an exploratory analysis. With several predictors and only 45 observations, the model is also likely overfitted. This is particularly important because the manuscript presents R^2 and partial R^2 values in a way that may imply more certainty than the data can support.

Thank you very much for your comment. We agree that the 45 clinical-study estimates cannot be regarded as fully independent, because they were derived from only 10 carbon-footprint assessments and may share methods, assumptions, emission factors, institutions, or sponsors within each assessment. **We therefore revised the statistical analysis to account for the clustering of clinical studies within carbon-footprint assessments.** Confidence intervals and P values are now estimated using CR2 cluster-robust variance estimation with a Satterthwaite small-sample correction. We also reduced the number of variables in the model from [8] to 6, prioritizing clinical study characteristics considered emission wise more relevant. **The updated regression results are presented in Table 4.** Following these revisions, none of the examined associations was statistically significant, and the confidence intervals were generally wide.
The regression is now explicitly described throughout the manuscript as exploratory, and the adjusted R^2 and partial R^2 values are interpreted only as descriptive measures of variation. We have reviewed the entire manuscript to
</td></tr>
</table>

	make sure removed language presenting countries, sites, study design, or assessment method as established drivers or independent predictors and instead describe them cautiously as potential sources of measurement variation. We also acknowledge that, despite the small-sample correction, the limited number of assessment-level clusters continues to constrain statistical precision and interpretation.
2	A second major issue concerns the quality-weighted mean of 355.3 tCO_2-eq per clinical study. While this is an interesting descriptive estimate, its interpretation is problematic because the underlying studies differ greatly in design, scale, country coverage, trial phase, system boundaries, emission factors, and inclusion of intervention manufacturing. Weighting by a custom quality score may not solve this heterogeneity and may even create a false sense of methodological precision. I suggest presenting this estimate more cautiously, emphasizing the median and range, and using sensitivity analyses excluding extreme values, industry-sponsored trials, early-phase pharmaceutical trials, and studies with incomplete activity disaggregation.<br><br>**Thank you for this comment. We agree that weighting quality score does not address the substantial heterogeneity across clinical studies. We therefore removed the quality-weighted mean of 355.3 tCO_2-eq from the manuscript.** The revised Abstract, Results, and Discussion emphasize the range and median, while the unweighted mean and SD are retained in the Results only to describe the distribution and are not interpreted as representative estimates.<br><br>**In this revision, we expanded the stratified descriptive synthesis in Table 3 to present estimates according to geographical scale, study design, carbon-footprint assessment method (LCA-based or non-LCA-based), and quality category.** Table 3 also includes an analysis of the 11 pharmaceutical trials for which drug-production emissions were separately quantifiable. Table S-3 provides study-level carbon-footprint estimates with and without drug-production-related emissions for the pharmaceutical trials.<br><br>**We believe that many other exclusion/sensitivity analyses would have limited interpretability given the heterogeneous evidence base, particularly the variation in carbon-assessment methods and substantial overlap among the proposed exclusion/sensitivity criteria.** Excluding studies according to these characteristics would produce small and selective subsets, and differences between the resulting subsets could not be clearly attributed to the characteristic used for exclusion rather than to other methodological differences. **In the Discussion section we added:**<br>*Within this limited evidence base, study design also overlapped with sponsor type, trial phase, geographical scale, and the inclusion of intervention-manufacturing emissions, making these influences difficult to separate.”*
3	The manuscript would also benefit from clearer distinction between variability caused by true operational differences and variability caused by carbon-accounting methodology. For example, higher emissions in LCA-based studies may reflect more complete capture of emission sources rather than genuinely more carbon-intensive research. This distinction should be made explicit throughout the

	results and discussion. Similarly, the finding that RCTs have higher emissions than pragmatic trials may be confounded by sponsor type, trial phase, intervention manufacturing, and international scale. The authors should avoid implying causality from these associations.<br><br>**We agree that variation arising from clinical study characteristics and conduct should be distinguished from variation arising from carbon-accounting methodology. We have made this distinction explicit throughout the manuscripts:**<br>The revised Discussion states:<br>*"The observed variation in carbon-footprint estimates may reflect, on the one hand, differences in clinical study characteristics and conduct and, on the other, methodological differences in carbon-footprint assessment. These sources of variation could not be fully disentangled in the available evidence. Descriptive summary measures were higher in LCA-based than in non-LCA assessments, and the use of an LCA-based assessment accounted for the next-largest additional proportion of variation in the multivariable model. This difference could reflect either the broader system boundaries and more comprehensive coverage of emission sources commonly used in LCA-based assessments or genuinely greater carbon intensity among the clinical studies assessed using these methods."*<br><br>**We have applied the same caution to the comparison of RCTs and pragmatic trials.** The revised Discussion states:<br>*"Similarly, descriptive summary estimates were higher for RCTs than for pragmatic trials; however, the available evidence was too limited to draw conclusions about an independent effect of study design. Within this limited evidence base, study design also overlapped with sponsor type, trial phase, geographical scale, and the inclusion of intervention-manufacturing emissions, making these influences difficult to separate."*<br><br>**In the revised Results, differences according to assessment method and study design are presented as descriptive findings. The multivariable analysis is explicitly described as exploratory.** We therefore refer to the examined characteristics as potential sources of variation rather than established drivers of emissions. **We reviewed the manuscript for removing language implying causal or independent effects.**
4	There appears to be an inconsistency in the reporting of the regression results for randomized controlled trials. The abstract reports β=0.97 with 95% CI 0.68–1.27, whereas the results/table appear to show β=0.97 with a confidence interval that is either incorrectly ordered or inconsistent. This must be corrected. All regression estimates, confidence intervals, p values, and interpretations should be checked carefully.<br>**Thank you for this important comment. We reviewed all regression reporting throughout the manuscript against the updated statistical output and revised the coefficients, 95% confidence intervals, P values, and associated interpretations in the Abstract, Results, and Table 4.** We also cross-checked all reported estimates against the final output to ensure consistency.
5	The quality appraisal tool is useful but requires further justification. Because it is customized and not externally validated, the authors should explain how the

	domains were selected, whether the scoring system was piloted, whether inter-rater agreement was assessed, and how subjective classifications such as hospital resource demand were handled. The high/medium/low hospital resource demand classification is potentially valuable, but the criteria need to be more transparent and reproducible. It would help to provide examples and explain how disagreements were resolved.<br>**Thank you for this comment. We have expanded the Methods to clarify the development and application of the customized quality-appraisal checklist.** The domains were informed by guidance on sustainable care pathways and international life cycle assessment standards (ISO 14040/14044) and developed iteratively through consensus discussions between an environmental and life cycle assessment expert and a clinical epidemiologist and methodologist. In the absence of an empirical basis for differential weighting, all domains were equally weighted, and the scoring criteria and thresholds are now reported explicitly. **The complete checklist, more details about the method, domain definitions, and scoring criteria are provided in Methods S-4 and Table S-1.**<br><br>**Formal piloting and external validation of the checklist, including its classification thresholds, were beyond the scope of this review.** We did not calculate a quantitative measure of inter-rater agreement; however, all assessments were appraised independently by two reviewers, with discrepancies resolved through discussion and consensus we elaborated in the method of the manuscript and the supplementary materials together with the checklist. **We therefore include a section as limitations and recommendation for future research.**<br><br>*"The tailored quality-appraisal checklist and its classification thresholds were not formally piloted or externally validated. Although assessments were appraised independently by two reviewers and discrepancies were resolved through consensus, inter-rater agreement was not quantified. Formal evaluation of the checklist's validity and reliability, would therefore be a valuable area for future research."*
6	The hotspot analysis is one of the strongest and most practice-relevant parts of the paper, but it should be strengthened by explaining how activities were harmonized when studies used different categories. Some studies did not report disaggregated emissions, and this missingness could bias the hotspot findings. The authors should state whether hotspots were calculated only among studies with available disaggregation or whether missing categories were treated in another way.<br><br>**Thank you for this comment. We have expanded the Methods section to elaborate the steps of hotspot analysis with more details.** Activity-specific emissions reported using different classifications were harmonized by mapping activities, based on their descriptions, to ten predefined groups adapted from Griffiths et al. **As shown in Table S-5, a hotspot was assigned to each of the 45 clinical studies** based on the highest-emitting activity among those reported. However, where activity-level reporting was incomplete, missing activity groups were coded as N/A rather than as zero; consequently, the assigned hotspot may have been influenced by incomplete reporting. Which has been mentioned in the interpretation of the results .

	Results: "*Table S-5 summarizes the distribution of CO_2-eq emissions across predefined activity groups* ***for the 45 clinical studies****. A hotspot was assigned to each study based on the highest-emitting activity among those reported. Meetings and travel (the highest-emitting activity in 17 studies) and clinical study unit operations (the highest-emitting activity in 13 studies) most frequently emerged as emission hotspots. These patterns were most commonly observed in studies involving a larger number of sites and/or multiple countries.* ***However, activity-level reporting was incomplete in several studies, particularly those using non-LCA approaches. Unreported activity groups were coded as not available rather than as zero; consequently, for these studies, the assigned hotspot represents the highest-emitting activity among those reported and may have been influenced by incomplete disaggregation****.*"
7	The discussion is generally thoughtful, but it should be more balanced. The manuscript sometimes moves from descriptive evidence to broad recommendations for governance, regulation, and research training without fully acknowledging the limited empirical base. These recommendations are reasonable, but they should be framed as implications requiring further development rather than direct conclusions from the current dataset. The authors should also expand the equity perspective. Most included evidence comes from high-income settings, and the carbon implications of research in low- and middle-income countries may differ substantially because of infrastructure, travel needs, decentralised care models, regulatory requirements, and capacity constraints.<br><br>**Thank you very much for this feedback and comment.** We have revised the Discussion to distinguish these broader implications from the empirical findings and to frame them as areas requiring further methodological development and evaluation rather than as direct conclusions of this review.<br><br>***Discussion section: "****These findings, however, are based predominantly on evidence from high-income settings. Emission profiles and feasible mitigation strategies may differ in low- and middle-income settings because of differences in research infrastructure, energy systems, travel requirements, decentralised care models, regulatory processes, and capacity constraints. Mitigation approaches developed in high-income settings should therefore not be assumed to be directly transferable and should be adapted without shifting burdens to participating sites or undermining capacity strengthening and equitable research participation.***"***<br><br>***Discussion section: "****However, the broader implications for research governance, reporting standards, training, legislation, and regulation extend beyond the evidence directly examined in this review and should be regarded as areas for further methodological development and empirical evaluation. Within this broader agenda, carbon accountability could also extend to research dissemination, as emissions associated with unpublished studies may represent avoidable environmental waste. Clinical researchers may have an important role through decisions made during study design and conduct, but responsibility would also extend to methodologists, institutions, regulators, and guideline developers. Future work should examine how environmental considerations can be incorporated into these structures without compromising scientific validity, participant safety, or equity.***"***

8	The authors should consider citing additional recent literature on sustainability, public health, and implementation of sustainable health-related practices where appropriate. Relevant works that could strengthen the broader discussion include 10.62210/ClinSciNutr.2024.93, because it discusses the integration of sustainability-oriented approaches into clinical care and could help connect the proposed carbon-accounting framework with practical clinical implementation; 10.1007/s11356-023-27694-8, because it links food insecurity with sustainable and healthy behaviours and is relevant to the manuscript's equity-oriented discussion of sustainability in health systems; 10.3389/fnut.2025.1684438, because it addresses knowledge-to-action pathways for sustainable health behaviours, which is relevant to translating carbon-footprint evidence into behavioural and institutional change; and 10.3389/fpubh.2025.1569755, because it highlights global vulnerability patterns across diet, health, and health-system indicators, which would support the authors' acknowledgement that most current evidence comes from high-income settings. These citations should not be added superficially, but they are relevant if the authors expand the discussion around sustainability implementation, health equity, and public-health relevance.<br><br>Thank you for suggesting these additional references. We carefully reviewed each of them when revising the broader discussion of implementation, equity, and public-health relevance. These studies provide valuable perspectives on:<br><br>- plant-based nutrition in clinical care (doi:10.62210/ClinSciNutr.2024.93);<br>- food insecurity and sustainable and healthy eating behaviours (doi:10.1007/s11356-023-27694-8);<br>- nutrition knowledge, sustainable eating, and Mediterranean diet adherence (doi:10.3389/fnut.2025.1684438); and<br>- global vulnerability patterns across diet, health, and health-system indicators (doi:10.3389/fpubh.2025.1569755).<br><br>**However, clinical care and general healthcare activities were distinguished from research-related activities wherever possible in our review. Nutrition was not identified as a research-related activity in any of the included carbon-footprint assessments or relevant frameworks. <u>We therefore considered that a detailed discussion of these studies would extend beyond the current focus of our review.</u>** We have therefore recommended that future research explore whether and how these specific factors should be incorporated into the scope of future research/discussions<br>We added the following sentences to the Discussion paragraph addressing assessment scope:<br>*" Similar questions of assessment scope may apply to other activities. Food- and nutrition-related emissions were not considered in the carbon-footprint assessments included in this review or in the activity framework proposed by Griffiths et al(4). However, previous studies have considered these emissions in carbon-footprint assessments of care pathways. Future research should explore whether and under what circumstances they should be included within the scope of carbon-footprint assessments of clinical research."*
9	The language is mostly understandable, but the manuscript requires careful editing for grammar, repetition, and precision. For example, there are repeated phrases in the methods, some awkward wording such as "this variation affect comparability,"

	and inconsistent use of "trial," "clinical study," and "clinical research." The authors should standardize terminology and ensure that "clinical trial" is not used when the included evidence also covers broader clinical research.<br><br>Thank you for this comment. We carefully reviewed the manuscript for grammar, repetition, precision, and consistency of terminology. Throughout the manuscript, the terminology has been harmonized. **"Clinical research" or "clinical study" refer to the overall subject examined in this review and is defined as health or healthcare research involving human participants that investigates health, disease, or healthcare. They used for each underlying study serving as a unit of analysis in the quantitative synthesis. "Carbon-footprint assessment" used for an included publication that estimated the emissions of one or more clinical studies.**
10	Overall, this is a valuable and potentially publishable manuscript, but the current version overstates some quantitative conclusions and needs stronger methodological caution, clearer handling of clustering and heterogeneity, corrected regression reporting, improved explanation of the quality appraisal and resource-demand classification, and a more balanced discussion of generalizability.<br><br>Thank you for this overall assessment. As detailed in our point-by-point responses, we have revised the manuscript to interpret the quantitative findings more cautiously, account for clustering in the multivariable analysis, address heterogeneity more explicitly, correct and verify the regression reporting, clarify the development and application of the quality-appraisal tool and hospital resource-demand classification, and provide a more balanced discussion of generalizability across research settings.

<table>
<tr><td colspan="2">

Reviewer: 3

Dr. Peter Doran, University College Dublin School of Medicine and Medical Science, University College Dublin
Comments to the Author:

This is a timely and relevant systematic review addressing an increasingly important topic: the environmental impact of clinical research. The manuscript is a valuable addition to the literature and has a number of strengths, including:
• A clearly defined research question.
• Systematic review methodology with PROSPERO registration.
• An attempt to move beyond narrative synthesis by incorporating quantitative analyses
• Practical implications for trial design and sustainability policy.
However , I would suggest the authors further consider some specific methodological and reporting issues.

Thank you, Dr Doran, for your comments and for highlighting the relevance and strengths of our systematic review. We carefully considered the methodological and reporting issues raised and revised the manuscript accordingly. Our point-by-point responses are provided below.
</td></tr>
<tr><td>1</td><td>Limited evidence base versus strength of conclusions
The review includes only 10 studies representing 45 primary clinical studies, and most originate from high-income countries and a relatively small number of research groups. The conclusions regarding average emissions, determinants of emissions, and hotspot patterns may therefore appear stronger. The authors should emphasise more strongly that the field remains highly immature. In particular the should frame the reported quality-weighted mean (355.3 tCO_2-eq) as a provisional estimate rather than a benchmark.

Thank you very much for this comment. We agree with the reviewer and therefore removed the quality-weighted mean from the manuscript and now emphasize the median, interquartile range, and overall range of emissions. The unweighted mean and standard deviation are retained only as descriptive measures and are not presented as representative estimates.

We expanded the Strengths and limitations section to discuss the limited number of carbon-footprint assessments, the concentration of evidence in high-income settings and among a small number of research groups, and the resulting constraints on independence, representativeness, and statistical precision.</td></tr>
<tr><td>2</td><td>Quality appraisal tool requires stronger justification and validation
The review uses a customized 8-domain quality appraisal tool developed by the authors. While understandable and highly interesting given the lack of existing tools, the manuscript provides limited evidence that the instrument is reliable or validated. This generates some concerns including that the Domain weighting appears arbitrary, Equal weighting of all domains may not reflect their importance and the rationale for the cutoffs (high/medium/low quality) is unclear.
I would suggest that the authors expand the discussion to provide more detail on the development process of the tool, the pilot testing, inter-rater reliability measures and perhaps consider including sensitivity analyses showing if results change when quality weighting is omitted.</td></tr>
</table>

	Thank you for your comment. We expanded the Methods to describe the development and application of the customized quality-appraisal checklist. The domains were informed by guidance on sustainable care pathways and international life cycle assessment standards (ISO 14040/14044) and were developed iteratively through consensus discussions between an environmental and life cycle assessment expert and a clinical epidemiologist and methodologist. The complete checklist, domain definitions, scoring criteria, and classification thresholds are provided in the supplementary material. We consider the development and application of the checklist through this structured process valuable for the current review; however, **its formal evaluation warrant dedicated future research.**<br><br>**In the absence of an empirical basis for assigning different weights, the eight domains were equally weighted.** The equal weighting and the thresholds used to classify assessments as high, medium, or low quality have not been empirically validated. The checklist was not formally piloted or externally validated, and inter-rater agreement was not quantified. **Formal validation and reliability assessment were beyond the scope of the current review. These points are now explicitly acknowledged in the limitations section, where we also recommend formal evaluation of the checklist's validity and reliability in future research.**<br><br>In response to the concern regarding quality weighting, we removed the quality-weighted emission estimate from the manuscript. Emission estimates are now presented using unweighted descriptive measures and are additionally stratified by quality category, without using the quality score to weight the quantitative synthesis.
3	Statistical modelling<br>The multivariable regression includes multiple predictors despite habving just 45 observations which may introduce overfitting. The authors should report more details including Variance inflation factors (VIFs), Residual plots and ensitivity analyses using simpler models.<br><br>Thank you for this comment. We agree that the number of observations and assessment-level clusters limits the precision of the multivariable analysis estimates. **Given the limited sample size, the analysis is now consistently described as exploratory. We therefore reduced the model from eight to six predictors (variables), prioritizing contextually relevant variables**. To account for the clustering of clinical studies within carbon-footprint assessments, **confidence intervals and P values are now estimated using CR2 cluster-robust variance estimation with a Satterthwaite small-sample correction**. Additionally, we **assessed multicollinearity using generalized variance inflation factors; adjusted GVIFs** ranged from 1.28 to 2.25, implying no evidence of substantial multicollinearity.<br><br>Overall, despite these additional analytical considerations, the limited number of observations and clusters remains an important constraint, and the regression estimates, adjusted R^2, and partial R^2 values are interpreted cautiously as exploratory.
4	Interpretation of causal relationships<br>There are a number of points at which discussion statements imply causal

	mechanisms – For example more countries → higher emissions…. more sites → higher emissions… RCTs → higher emissions<br>While these observations are plausible and likely to be born out, the study design is observational and cross-sectional. The analyses demonstrate association rather than causation. I would encourage the authors to use more cautious language such as "associated with" , "may contribute to" and "consistent with the hypothesis that" rather than implying direct causality.<br><br>**Thank you for your comment. We reviewed the manuscript including the Abstract, Results, Discussion, and Conclusion to make sure we removed language implying causal or independently established effects.**<br>Descriptive differences are reported as patterns in carbon-footprint estimates, while the examined characteristics are discussed as potential sources of variation. We also clarify that the multivariable analysis was exploratory<br>Accordingly, the numbers of participating countries and study sites are described as aspects of geographical scale that may contribute to variation in carbon-footprint estimates, while the proposed logistical mechanisms are presented only as possible explanations for the observed patterns.<br><br>Differences between RCTs and pragmatic trials are reported descriptively, with explicit acknowledgement that study design overlapped with sponsor type, trial phase, geographical scale, and inclusion of intervention-manufacturing emissions. We therefore think that we draw no causal conclusions from these findings.
5	Heterogeneity in carbon accounting methods<br>The authors show a central finding is that methodological variation drives much of the observed variability. I think this could be explored in more detail, perhaps including a discussion of …<br>• How much variation is attributable to methodology versus trial characteristics?<br>• Are LCA-based studies systematically higher because they capture more sources?<br>Whilst these are in many ways complex questions, a really nice addition to the manuscript would be A table comparing carbon-accounting approaches with a sensitivity analysis stratified by accounting method.<br><br>**Thank you for this comment. In response to your comment we expanded the descriptive synthesis to stratify carbon-footprint estimates by assessment method, with LCA-based and non-LCA-based assessments compared in Table 3. Descriptive summary estimates were higher for LCA-based assessments. In the exploratory multivariable analysis, assessment method accounted for an additional 9.8% of variation conditional on the other included variables, although the association was not statistically significant and remained uncertain.**<br>We also expanded the Discussion to distinguish variation arising from clinical study characteristics and conduct from variation arising from carbon-footprint assessment methods. Higher estimates from LCA-based assessments may reflect broader system boundaries and more comprehensive capture of emission sources; however, LCA-based methods were also used more frequently for larger, industry-led studies. Consequently, the respective contributions of assessment methodology and the characteristics of the studies being assessed could not be fully disentangled.

	**The characteristics and methods of the included carbon-footprint assessments are compared in Table 1, while carbon-footprint estimates stratified by assessment method are presented in Table 3.** Given the limited number of assessments and the overlap between assessment method and clinical study characteristics, these findings are presented descriptively and interpreted cautiously. We have made this limitation explicit and avoided attributing the observed differences solely to the carbon-accounting method.
6	hospital resource demand classification<br>The classification into low, medium, and high resource demand appears at least to my reading to be subjective. Can the authors provide explicit cirteria, with examples?<br><br>**Thank you for this comment. We therefore expanded the related Method section and Methods S-3 to define the classification according to the intensity of clinical care and healthcare infrastructure required, considering intervention type, diagnostic or therapeutic technologies, care setting, and level of clinical support. Explicit examples are provided for each category:** high-resource-demand studies included major surgery, radiotherapy, advanced imaging, and acute inpatient or intensive care; medium-resource-demand studies included chronic disease management, chemotherapy, minimally invasive procedures, and repeated outpatient assessments; and low-resource-demand studies included behavioural or lifestyle interventions, blood-donation studies, and outpatient pharmacological studies requiring minimal infrastructure. **Two reviewers independently classified each clinical study, with disagreements resolved through discussion and consensus.**

**Reviewer: 4**<br>Dr. William Gray, NHS Improvement<br>Comments to the Author:<br>This is a well written study, and the findings are of interest. I have a few comments.<br><br>**Thank you very much Dr. Gray for your comments and assessment of our study. We have carefully considered and addressed each of your comments, as detailed below.**	
1	I am confused by the description of 'study level' and 'primary clinical study level#. Could you say a bit more about this distinction. I assume it is to account for papers reporting multiple studies. Can I suggest picking one method and sticking to it to avoid confusion. I would have thought the primary clinical study level is the one to go for.<br><br>Thank you for raising this ambiguity. We have therefore standardized the terminology throughout the manuscript to distinguish two terms: the **"carbon-footprint assessment"** refers to the 10 included publications and their assessment characteristics, whereas the **"clinical study"** refers to the 45 underlying clinical studies for which emissions were estimated. The descriptive synthesis, hotspot analysis, and multivariable analysis were conducted at the clinical study level. We no longer use the ambiguous term "study level" or the term "primary clinical study."
2	The sentences below appear to broadly repeat the same information: "Primary clinical study characteristics include name, design, sample size, number of sites and countries, study duration (time between start and end dates). At the primary clinical study level, extracted characteristics included study name, design, sample size, number of sites and countries, and study duration (time between start and end dates)."<br>Thank you for your comment. We reviewed the manuscript to ensure that this information is stated only once and retained the following wording: *"At the clinical study level, extracted characteristics included study name, design, sample size, number of sites and countries, and study duration (time between start and end dates)."*
3	Statistical methods: I am not convinced of the need for the linear regression analyses or the weighting for quality. Both methods are likely to have methodological flaws. For linear regression, it would be good to see evidence of validity assessment (eigenvalues and vector analysis, VIF calculation etc). I would have thought multicollinearity was an issue and it would be good to know if it was. Do the independent variables meet all the assumptions of linear regression? With regard to the weighting. How do you know that this crude weighting method is valid? I don't think the scoring system, given that it is an ordinal scale, will have been designed to allow for weighting in this way. Should a study that scores 6 really have double the weighting of a study that scores 3? My feeling is that neither of these analyses add a huge amount and you could draw exactly the same conclusions with fewer caveats by simply reporting crude descriptive data. You don't need a regression model to point out that an RCT has a higher per patient carbon footprint than a cohort study.

	**Thank you very much for your comment. We agree that the quality score does not provide an empirically validated basis for weighting emission estimates. We therefore removed the quality-weighted mean and all quality-weighted analyses from the manuscript. Emission estimates are now reported using unweighted descriptive measures and are stratified by quality category only for descriptive comparison.**<br><br>We retained the multivariable regression as an exploratory analysis because it allows study characteristics to be examined conditionally on the other included variables, which cannot be achieved through crude descriptive comparisons alone. We think we substantially revised and downgraded its role in interpretation and in Discussion The model is now reduced from eight to six predictors, and confidence intervals and P values were estimated using CR2 cluster-robust variance estimation with a Satterthwaite small-sample. Multicollinearity has now been assessed using generalized variance inflation factors; adjusted GVIFs ranged from 1.28 to 2.25, suggesting no evidence of substantial multicollinearity.<br>Following these revisions, none of the examined associations was statistically significant, and confidence intervals were generally wide. The descriptive synthesis is therefore given greater prominence, while the regression estimates, adjusted R^2, and partial R^2 values are interpreted cautiously as exploratory measures of conditional variation. These changes are highlighted in yellow in the revised manuscript.
4	Related to the above point, the authors report and discuss the quality weighted average (355 tonnes CO2e, but place less emphasises on the crude average (mean or median), which I don't think is mentioned to any great extent. This value is perhaps the more interesting one. In lieu of using the quality weighted average, you could report the average for studies meeting and not meeting a given quality score or graph how values of CO2e vary across the quality scores. This may be of more value to readers and make a simpler and more digestible point.<br><br>**Thank you for this comment. We removed the quality-weighted average from the manuscript and now give greater prominence to the unweighted descriptive estimates**. Because the distribution was strongly right-skewed, we emphasize the median of 78.0 tCO_2-eq per clinical study (IQR 54.9–171.0) and the overall range of 8.8–3,107 tCO_2-eq. The unweighted mean of 341.5 tCO_2-eq (SD 658.8) is also reported as a descriptive measure but is not presented as representative of a typical clinical study.<br>**As suggested, Table 3 now presents unweighted mean, SD, median, and IQR values stratified by quality category, allowing direct comparison between emission estimates from high- and medium-quality assessments**. No assessments were classified as low quality, and no clear descriptive pattern in carbon-footprint estimates was observed across the available quality categories.
5	Was the outcome of interest total carbon emissions for the entire study, or per patient carbon emissions? Per patient makes more sense.<br><br>**Thank you for this comment. The outcome of interest in the quantitative synthesis and multivariable analysis was the total carbon footprint of conducting an entire clinical study.** This outcome aligns with the review objective and captures study-level emissions, including coordination,

	infrastructure, monitoring, travel, and other activities that cannot necessarily be attributed to individual participants.<br>We additionally reported emissions per participant descriptively where sample-size information allowed this calculation. However, the available assessments did not provide participant-level emission data disaggregated across sites, countries, or study activities. Moreover, dividing total emissions by sample size would distribute fixed study-level emissions equally across participants and could make larger studies appear more efficient without reflecting differences in their overall footprint. We have clarified in the Methods that total study-level emissions were used as the regression outcome.
6	The hotspot analysis adds value as it can lead to an informed discussion of mitigation, although the message seems to be that the hotspots are very study specific. It should perhaps be more of a focus.<br><br>**Thank you for this comment. We have given greater emphasis to the hotspot analysis throughout the revised manuscript.** Specifically, we moved the hotspot analysis earlier in both the Methods and Results, before the exploratory regression analysis, to better reflect its importance as a primary descriptive analysis. We also expanded the Discussion of the hotspot findings and their implications for mitigation. In particular, we now highlight that, although some activity groups were recurrently identified as major emission sources, the dominant sources varied across clinical studies. This suggests that mitigation strategies may need to be tailored to the activities contributing most to the carbon footprint of individual studies rather than assuming a single dominant source across clinical research.
7	"Use of a life cycle assessment (LCA)-based carbon calculation system was marginally statistically significant and associated with higher estimated emissions compared with non-LCA approaches ($\beta = 0.97$; 95% CI: 0.00 to 1.96; $p = 0.05$)." This finding is difficult to interpret and raises the question as to whether it should be included in the model and the model design. The finding may just show that LCA methods estimate higher emissions or is it some (unaccounted for in modelling) characteristics of studies that used LCA. Most of the other independent variables seem to be design properties of the study, but this is not and seems out of place.<br><br>Thank you for this important comment. We retained carbon-footprint assessment method in the exploratory model because the reported emission estimate may depend not only on clinical study characteristics but also on how emissions were assessed. Including assessment method therefore allowed the relationships of clinical study characteristics with reported emissions to be examined while accounting, to some extent, for this methodological source of variation.<br>However, we agree that the estimate for assessment method cannot establish whether LCA-based approaches yield higher values because they capture emissions more comprehensively or because they were applied to intrinsically more carbon-intensive studies. LCA-based assessments were used more frequently for larger, industry-led studies, and these influences could not be fully disentangled in the available data. We have made this distinction explicit in the Discussion and made sure we did not interpret the assessment-method as an independent or causal effect.

	Following revision of the model and application of cluster-robust inference with a small-sample correction, assessment method was no longer statistically significant. It is now presented as a potential methodological source of variation and accounted for the third-largest additional proportion of variation in the exploratory model (partial R^2=9.8%), with appropriate caution regarding its interpretation.
8	The discussion is well written. It has large sections on hotspot mitigation, which is good, but emphasises that this should be more of a focus of the results section.<br><br>Thank you for this comment. We have given the hotspot analysis greater prominence in the revised Results by placing it before the exploratory regression analysis and expanding its presentation. The revised section now reports the frequency with which each activity group was identified as the dominant emission source and describes how hotspot patterns differed according to geographical scale and hospital resource demand. We also clarify that activity-level reporting was incomplete in several studies and that, in these cases, the assigned hotspot represents the highest-emitting activity among those reported. The full activity-level breakdown and assigned hotspot for each of the 45 clinical studies are provided in Table S-2.
9	I agree on the need for a guiding framework on scope and boundaries for clinical studies. Such guidance is much needed to ensure consistence of reporting. The authors may wish to expand on this point. I know there are groups in the UK (e.g. Grener Trials) with an interest in this area. This point could also be linked to the paragraph immediately after on variability.<br><br>Thank you for this comment. We expanded the Discussion of assessment scope and system boundaries and linked it directly to the subsequent discussion of methodological variability. The revised text explains that a standardized and transparent framework should provide guidance on defining system boundaries, selecting and grouping emission sources, choosing emission factors, attributing shared activities, handling assumptions and missing data, conducting uncertainty analyses, and reporting estimates. We also emphasize that such a framework should improve consistency and comparability while remaining adaptable to different study designs, interventions, clinical fields, and settings.<br>**We now acknowledge relevant existing initiatives, including the UK Greener Trials group, whose work may provide a useful basis for further methodological development and collaboration in this area. The relevant references have also been added to the manuscript.**
10	One point the authors could pull out more in the discussion section early on is that the mean/median emissions of a trial are relatively high (355 tonnes CO2e). The authors may want to offer a comparison of what this means in terms of car journeys, hot meals or heating, just to place it into perspective. They could also compare it to routine clinical care, where some useful estimates exist.<br><br>Thank you for this suggestion. We agree that the magnitude of emissions deserves greater emphasis and have brought this point forward in the Discussion while also highlighting the substantial variation across clinical studies. As described in response to the comments above, we removed the quality-weighted mean of 355.3

	tCO_2-eq and now emphasize the median, interquartile range, and overall range rather than presenting a single value as representative.<br><br>We considered expressing these estimates using equivalents such as car journeys, meals, or household heating. However, such conversions depend strongly on the selected scenario, geographical context, and emission factors and could introduce another layer of assumption-dependent comparison. We also considered comparisons with routine clinical care, but the available estimates often use different functional units, system boundaries, and attribution rules. Direct comparison could therefore conflate emissions attributable to research with those arising from care delivery. **We consequently chose not to include these comparisons and instead describe the emissions as considerable while retaining the observed distribution and methodological uncertainty needed to interpret that conclusion.**
11	References 1, 7 and 27 appear wrongly and need to be corrected. I think you have used Endnote and not put a common after the entire organisation's name (e.g. World Health Organisation,)<br><br>Thank you for identifying this issue. We corrected the EndNote records for references 1, 7, and 27 to ensure that the organization names are formatted correctly as corporate authors. We also checked the remaining references and corrected similar formatting issues where identified.

VERSION 2 - REVIEW

**Reviewer**	**2**
**Name**	**Arslan, Sedat**
**Affiliation**	**Bursa Uludag University**
**Date**	**30-Aug-2026**
**COI**	

Dear Authors,

Thank you for your careful and comprehensive revision. The manuscript has improved substantially, and the major methodological concerns raised in the previous review have been appropriately addressed. In particular, the revised analysis now accounts for clustering of clinical studies within carbon-footprint assessments, the regression is clearly framed as exploratory, and the interpretation of adjusted and partial R^2 values is more cautious. The removal of the quality-weighted mean and the greater emphasis on the median, interquartile range, and overall range also improve the interpretation of the heterogeneous evidence base.

The distinction between variation related to clinical study characteristics and variation arising from carbon-accounting methodology is now much clearer. The revised discussion appropriately avoids causal interpretation of differences by study design or assessment method and acknowledges the limited ability to disentangle these factors.

The additional clarification of the customized quality-appraisal tool, hospital resource-demand classification, and hotspot analysis has also strengthened the methodological transparency of the manuscript. The limitations relating to the lack of formal validation and inter-rater reliability assessment are now appropriately acknowledged.

The discussion is now more balanced regarding generalizability, particularly in relation to low- and middle-income settings and the implications for governance and implementation.

Only minor editorial issues remain. I recommend a final careful check of table and supplementary-table numbering in the manuscript and response document, together with a final language and terminology review for consistency.

Overall, the manuscript has been substantially strengthened and I am satisfied that the main concerns from the previous review have been addressed.

**Reviewer**	**3**
**Name**	**Doran, Peter**
**Affiliation**	**University College Dublin School of Medicine and Medical Science, School of Medicine**
**Date**	**31-Aug-2026**
**COI**	

I thank the authors for their careful attention to all of the reviewer comments. I believe this is now a substantially strengthened manuscript that adds significantly to the literature.

**Reviewer**	**4**
**Name**	**Gray, William**
**Affiliation**	**NHS Improvement, Getting It Right First Time**
**Date**	**15-Sep-2026**
**COI**	

The manuscript is much improved. The presentation of the findings is now more balanced and reflects the nature of the data. I have no additional comments.
