# Supplementary material for: Carbon footprint of clinical research: a systematic review and quantitative synthesis of estimates, sources of variation and emission hotspots
Source: BMJ Open. 2026 Oct 5;16(10):e122205. doi: 10.1136/bmjopen-2026-122205 (PMC13646982; doi:10.1136/bmjopen-2026-122205)
Supplement: Supplementary file 2 [file bmjopen-16-10-s001.docx]

**Supplementary Materials**

**The Carbon Footprint of Clinical Research Practice: Synthesizing Current Evidence Calls for Standardized Assessment Frameworks**

Mitra Nekouei Shahraki, MD, PhD*(1, 2); Lisa van Bodegraven, MSc (1, 2); Florine de Haes, MSc (2); Anneke Kwee, MD, PhD (3, 4); Nicolaas H. Sperna Weiland, MD, PhD (2, 5); Wouter .J.K Hehenkamp, MD, PhD (2, 6); Mariska M.G Leeflang, PhD (1); Dorien A Salentijn, MD (1, 2, 7)

(1) Department of Epidemiology and Data Science, Amsterdam University Medical Center, Amsterdam, the Netherlands

(2) Center for Sustainable Healthcare, Amsterdam University Medical Center, Amsterdam, the Netherlands

(3) Program Health Care Evaluation and Appropriate Care, Health Care Institute Netherlands, Diemen, the Netherlands

(4) Department of Fertility, Gynaecology, and Obstetrics, OLVG hospital, Amsterdam, The Netherlands

(5) Department of Anesthesiology, Amsterdam University Medical Center, Amsterdam, The Netherlands

(6) Department of Obstetrics and Gynecology, Amsterdam University Medical Center, Amsterdam, The Netherlands

(7) Department of Radiology and Nuclear Medicine, Amsterdam University Medical Center, Amsterdam, The Netherlands

**Contents:**

- **Methods S-1:** Eligibility criteria
- **Methods S-2:** Search Strategy by Database
- **Figure S-1**: Prisma Flow Diagram
- **Methods S-3:** Categorization of hospital resource demand
- **Methods S-4:** Development and application of the quality-appraisal checklist
  - **Table S-1:** Quality-appraisal domains and scoring criteria for carbon-footprint assessments of clinical studies
- **Table S-2.** Characteristics of included carbon-footprint assessments and clinical studies, with reported CO₂-eq emission estimates
- **Table S-3:** Inclusion and contribution of drug-manufacturing emissions to carbon-footprint estimates across primary clinical studies
- **Table S-4:** Sensitivity analysis of carbon-footprint estimates with and without drug-production-related emissions in pharmaceutical trials
- **Table S-5:** Assessment of multicollinearity in the multivariable regression model
- **Table S-6:** Identification of emission hotspots: distribution of CO₂-eq emissions across ten predefined activity groups in 45 clinical studies
- **Table S-7:** PRISMA reporting checklist for the systematic review
- **Table S-8:** Potentially relevant studies excluded from this systematic review and reasons for exclusion

**Methods S-1:** Eligibility criteria

**Inclusion criteria**
Studies were included if they were:

1. Original full research articles
2. Estimated the carbon footprint potential in terms of quantified carbon dioxide equivalent (CO₂-eq) emissions associated with the conduct of patient-involved health research.

- This could be, for example, CO₂-eq estimates emitted by direct human activities related to research conduct (e.g., transportation of staff, patients, and equipment; energy consumption such as electricity or heating; material use such as laboratory samples or printed materials).

3. Were based on (or made an estimate using) any type of primary study design (as long as involving human subjects), including, for example, clinical trials (or pragmatic trials) and observational studies (cohort, case-control, cross-sectional, or qualitative research) of any sample scale, duration, funding source, country, or language.
4. Used any methodological approach for CO₂-eq calculation.

- This could be, for example, a Life Cycle Assessment

5. Were published at any time up to the search date

**Exclusion criteria**
Publications were excluded if they:

1. Did not originally quantify CO₂-eq emissions associated with the conduct of patient-involved health research.
2. Reported environmental impact metrics other than CO₂-eq, or did not provide sufficient information to calculate or derive CO₂-eq emissions.
3. Focused solely on non–patient-involved primary research (e.g., purely laboratory-based research).
4. Represented overlapping primary studies; in such cases, the publication evaluating the largest number of primary studies was included.

**Methods S-2:** Search Strategy by Database (the main stream search); November 2025

**Medline via OVID**

| **No.** | Ovid MEDLINE(R) ALL  **Search** | **Results** |
| --- | --- | --- |
| #1 | exp Clinical Trials as Topic/  OR exp Observational Studies as Topic/  OR exp Cohort Studies/  OR exp Case-Control Studies/  OR exp Cross-Sectional Studies/  OR exp Qualitative Research/ | 407764 |
| #2 | Carbon Footprint/ | 1413 |
| #3 | 1 and 2 | 7 |
| #4 | ((clinical or medical or biomedical or random*) adj2 (trial* or research or study or studies)).ti,ab,kf. | 1360391 |
| #5 | (((carbon or "CO2") adj3 (footprint or emission*)) or ((climate or environment*) adj impact)).ti,ab,kf. | 34061 |
| #6 | 4 and 5 | 290 |
| #7 | 3 or 6 | **274** |

**EMBASE**

| **No.** | **Query EMBASE**  **Search** | **Results** |
| --- | --- | --- |
| #7 | #3 OR #6 | **433** |
| #6 | #4 AND #5 | 44 |
| #5 | 'clinical trial (topic)'/exp  OR 'observational study'/exp  OR 'cohort analysis'/exp  OR 'case control study'/exp  OR 'cross-sectional study'/exp  OR 'qualitative research'/exp | 475286 |
| #4 | 'greenhouse gas emission'/exp | 22777 |
| #3 | #1 AND #2 | 258 |
| #2 | (((carbon OR 'co2') NEAR/2 (footprint OR emission*)):ti,ab,kw) OR (((climate OR environment*) NEAR/1 impact):ti,ab,kw) | 32106 |
| #1 | ((clinical OR medical OR biomedical OR random*) NEAR/2 (trial* OR research OR study OR studies)):ti,ab,kw | 1992516 |

**Web of Science (WoS)**

| **Entitlements** | **No.** | **Search Query** | **Database** | **Results** |
| --- | --- | --- | --- | --- |
| - WOS.SCI: 1975 to 2024 | #1 | Ti=((clinical or medical or biomedical or random*) near/2 (trial* or research or study or studies)) OR AB=((clinical or medical or biomedical or random*) near/2 (trial* or research or study or studies)) | Web of Science Core Collection | 1573951 |
| - WOS.AHCI: 1975 to 2024 |  |  |  |  |
| - WOS.ESCI: 2005 to 2024 |  |  |  |  |
| - WOS.SSCI: 1975 to 2024 |  |  |  |  |
| - WOS.SCI: 1975 to 2024 | #2 | Ti=(((carbon or "CO2") NEAR/3 (footprint or emission*)) or (climate-impact or environmental-impact) ) OR AB=(((carbon or "CO2") NEAR/3 (footprint or emission*)) or (climate-impact or environmental-impact) ) | Web of Science Core Collection | 220903 |
| - WOS.AHCI: 1975 to 2024 |  |  |  |  |
| - WOS.ESCI: 2005 to 2024 |  |  |  |  |
| - WOS.SSCI: 1975 to 2024 |  |  |  |  |
| - WOS.SCI: 1975 to 2024 | #3 | #2 AND #1 | Web of Science Core Collection | **497** |
| - WOS.AHCI: 1975 to 2024 |  |  |  |  |
| - WOS.ESCI: 2005 to 2024 |  |  |  |  |
| - WOS.SSCI: 1975 to 2024 |  |  |  |  |

**Figure S-1:** Prisma Flow Diagram

**Identification of studies via other methods**

**Identification of studies via databases and registers**

**Records identified (n=16):**

Reference and citation searching (n = 12)

LCA database (n = 3)

medRxiv and bioRxiv (n=1)

Records removed *before screening*:

Duplicate records removed (n = 444)

**Records identified (n=1204):**

Medline (n = 274)

Embase (n = 433)

WoS core collection (n = 497)

Total after deduplication (n=760)

**Identification**

Records screened

(n = 760)

Records excluded

(n = 738)

Reports not retrieved

(n = 1)

Reports sought for retrieval

(n = 16)

Reports not retrieved

(n = 1)

**Screening**

Reports sought for retrieval

(n = 22)

**Reports excluded (n=6):**

Full text unavailable (n = 1)

Wrong outcome (n = 4)

Overlapping primary study (n=4)

No original article (n = 4)

Reports assessed for eligibility

(n = 15)

Reports excluded:

Duplicates (n = 3)

Not eligible (n=10)

Reports assessed for eligibility

(n = 21)

**Reports included (n=6):**

Main stream search (n = 8)

Other sources (n = 2)

**Included**

**Methods S-3:** Categorization of hospital resource demand

We classified the hospital resource demand of primary clinical studies as high, medium, or low, reflecting the overall intensity of clinical care and healthcare infrastructure required. Classification was informed by the type of intervention, diagnostic or therapeutic technologies, care setting, and level of clinical support. High resource demand studies typically involved intensive or hospital-based care (e.g. advanced imaging, radiotherapy, major surgery, acute inpatient or intensive care settings). Medium resource demand studies generally required regular but non-intensive clinical involvement (e.g. chronic disease management, chemotherapy, minimally invasive procedures, repeated outpatient visits with laboratory or imaging assessments). Low resource demand studies involved limited clinical contact or community-based activities (e.g. behavioral or lifestyle interventions, health technology assessments, blood donation studies, or outpatient pharmacological studies with minimal infrastructure use).

**Methods S-4:** Development and application of the quality-appraisal checklist

The checklist assessed the methodological and reporting quality of CO₂-eq emission quantification in the conduct of clinical studies (patient-involved health research). It was developed drawing on existing guidance on sustainable care pathways and international life cycle assessment standards (ISO 14040/14044) [1-3].

The checklist was developed through rounds of consensus meetings between F.H. (environmental analyst, life cycle assessment expert, MSc) and M.N.S. (methodologist, epidemiologist, MD, MSc, PhD). It contains eight domains, described in the table below. Each domain was scored on a three-point scale: two points were assigned when the domain was fully met; one point was given when the domain was only partially met; and zero points were assigned when the domain was not met. The final quality score was calculated as the sum of domain scores.

**Table S-1:** Quality-appraisal domains and scoring criteria for carbon-footprint assessments of clinical studies

1. **Goal and scope clearly defined**

**2 points:** Unit of analysis defined as the complete scope of a clinical study (for example, from initiation of the study to the final closure and report) and assessed accordingly.

**1 point:** Unit of analysis defined but does not cover the complete scope (e.g., only part of the trial lifecycle is covered), or the unit of analysis is not clearly defined, or the assessment does not fully cover the defined unit.

**0 points:** Unit of analysis not specified.

2. **Scope coverage (completeness)**

**2 points:** Comprehensive coverage of the most common clinical study activities (relevant to the study), with all minimum essential activity categories included.

**1 point:** Some activities covered, but with some gaps, or insufficient justification for exclusions.

**0 points:** Very poor coverage of activities; major activities omitted without explanation.

3. **Activity data collection: transparent and reproducible**

**2 points**: Activity data collection fully transparent and reproducible, with a clear description of data collection methods.

**1 point:** Activity data collection reported but lacking some transparency or detail, reducing reproducibility.

**0 points:** Activity data collection unclear, with notable incompleteness and not reproducible.

4. **Activity data collection: real time**

**2 points:** Real-time data collection.

**1 point:** Retrospective data collection based on past records, or mixed with real-time data.

**0 points:** Prospective estimation of potential data (rather than observed activity), or data collection timing not reported or unclear.

5. **Using consistent standard emission factors**

**2 points:** Emission factors are consistently sourced from a single reliable source; calculations are reproducible.

**1 point:** Emission factors are sourced from different or multiple sources (less consistent); calculations are reproducible or partially reproducible.

**0 points**: Emission factors are sourced from unreliable or unknown sources.

6. **Assumptions clearly stated**

**2 points:** All methodological assumptions are explicitly described and justified.

**1 point:** Assumptions are incompletely described and justified.

**0 points:** Assumptions are not reported.

7. **Sensitivity or uncertainty analysis (accuracy)**

**2 points:** Sensitivity or uncertainty analysis conducted for important assumptions, missing data, or the use of surrogate data.

**1 point:** Limited sensitivity or uncertainty analysis performed, covering some but not all important testable assumptions, missing data, or surrogate data.

**0 points:** No sensitivity or uncertainty analysis performed, or no information provided on how important testable assumptions, missing data, or surrogate data were handled.

8. **Clear interpretation of findings and limitations**

**2 points:** All results interpreted clearly with explicit discussion of limitations and how they might affected the results.

**1 point:** Some results interpreted, and limitations partially addressed.

**0 points:** Results unclear or incomplete, and limitations not acknowledged.

**Table S-2.** Characteristics of included carbon-footprint assessments and clinical studies, with reported CO₂-eq emission estimates

| No. | Authors (year), institution | Primary Study N# | Name primary study, institution | Sample size | N# countries | N# sites | Study duration (years) | Hospital resource demand (high/medium/low) and specifications | Design | Carbon Calculation system- data collection direction | Estimated total CO₂-eq (tonnes) | Estimated CO₂-eq (tonnes)/ participants | QAR |
| --- | --- | --- | --- | --- | --- | --- | --- | --- | --- | --- | --- | --- | --- |
| **1** | **LaRoche et al.** (2025), by Johnson & Johnson Innovative Medicine, Delft, the Netherlands | 1 | Phase-1 trial, Janssen pharmaceutical | 39 | 1 (Belgium) | 1 | 1.25 | Low- antiviral drug as intervention (outpatient) | RCT | LCA- retrospective | 18 | 0.46 | H |
|  |  | 2 | Phase-2 trial Janssen pharmaceutical | 255 | 10 | 76 | N/A | Medium- drug for patients with psoriasis (outpatient) | RCT | LCA- retrospective | 580 | 2.27 |  |
|  |  | 3 | Phase-2 trial Janssen pharmaceutical | 125 | 13 | 127 | N/A | Medium- drug for urothelial cancer (outpatient) | RCT | LCA- retrospective | 1146 | 9.168 |  |
|  |  | 4 | Phase-3 trial Janssen pharmaceutical | 517 | 18 | 129 | N/A | High- drug effect and involved skeletal scans (CT, MRI, X-ray, outpatient) | RCT | LCA- retrospective | 3107 | 6.01 |  |
|  |  | 5 | Phase-3 trial Janssen pharmaceutical | 178 | 6 | 30 | N/A | Medium- drug (vaccine) in respiratory syncytial virus (RSV) diseases | RCT | LCA- retrospective | 359 | 2.02 |  |
|  |  | 6 | Phase-3 trial Janssen pharmaceutical | 1124 | 5 | 23 | N/A | Medium- drug effect and involved psychiatric assessments and labs | RCT | LCA- retrospective | 208 | 0.18 |  |
|  |  | 7 | Phase-4 trial Janssen pharmaceutical | 276 | 1  (India) | 11 | N/A | Medium- drug for glycemic control | RCT | LCA- retrospective | 171 | 0.62 |  |
| **2** | **Smit et al.** (2025), by Center for Infectious Diseases, Radboud University  Medical Center, the Netherlands | 8 | NECTAR3, phase 2 trial, London School of Hygiene and Tropical Medicine | 80 | 1 (Mali) | 1 | 0.25 | Low- antimalarial drug regimens in community / outpatient clinical setting | RCT | LCA- retrospective | 20.5 | 0.26 | H |
| **3** | **Cranley et al.** (2025), by Edinburgh Clinical Trials Unit, The University of Edinburgh, UK | 9 | COBALT trial, by Newcastle University | 300 | 1 (UK) | 25 | 3.75 | Low- drug for improvement of dementia and Parkinson’s disease dementia in outpatient clinical setting | RCT | Module-based carbon foot printing, Emission factor from Ecoinvent, frame work, retrospective | 26.3 | 0.09 | H |
|  |  | 10 | DASH Trial, by University of Nottingham | 54 | 1 (UK) | 10 | 4 | High- drug for spontaneous intracerebral hemorrhage, hospital-based, acute care | RCT | Module-based carbon foot printing, Emission factor from Ecoinvent, frame work, retrospective | 8.8 | 0.16 |  |
|  |  | 11 | Exenatide-PD3 trial by University College London | 194 | 1 (UK) | 6 | 5.5 | Low- drug for improvement of Parkinson’s disease in outpatient clinical settings | RCT | Module-based carbon foot printing, Emission factor from Ecoinvent, frame work, retrospective | 47.6 | 0.24 |  |
|  |  | 12 | LACI-2 trial by University of Edinburgh | 363 | 1 (UK) | 26 | 5 | Low- Drug for improvement of symptomatic cerebral small vessel disease in outpatient / community-based clinical setting | RCT | Module-based carbon foot printing, Emission factor from Ecoinvent, frame work, retrospective | 54.9 | 0.15 |  |
|  |  | 13 | PhEAST trial by University of Nottingham | 800 | 1 (UK) | 50 | 3 | High- device-based therapy for Post-stroke oropharyngeal dysphagia following acute stroke hospital-based patients | RCT | Module-based carbon foot printing, Emission factor from Ecoinvent, frame work, retrospective | 19.1 | 0.02 |  |
|  |  | 14 | RESTART trial by University of Edinburgh | 537 | 1 (UK) | 112 | 8 | Medium- drug for after recovery from intracerebral hemorrhage in hospital inpatients and outpatients clinical settings | RCT | Module-based carbon foot printing, Emission factor from Ecoinvent, frame work, retrospective | 106.4 | 0.20 |  |
| **4** | **Siegfried et al.** (2025), by South African Medical Research Council, South Africa | 15 | MIND trial by South African Medical Research Council | 1,340 | 1 | 24 | 4 | Low- task-shared psychological intervention in patients with chronic physical disease (HIV or type 1 or type 2 diabetes) outpatients setting | RCT | Activity-based carbon footprinting using the NIHR trial emissions framework, with a mix of South African-specific and UK emission factors, retrospective | 37.8 | 0.03 | H |
| **5** | **Griffiths et al.**(2024), by Clinical Trials and Statistics Unit, The Institute of Cancer Research, London, UK | 16 | EMERGE, phase-3 trial, National University of Ireland | 535 | 1 (Ireland) | 1 | 6 | Medium- drug for glycemic control in gestational diabetes mellitus | RCT | Emission factor from Ecoinvent, frame work, retrospective and prospective | 74 | 0.13 | M |
|  |  | 17 | HEAL-COVID trial, Cambridge University Hospitals | 1245 | 4 | 109 | 4.00 | Low- drug effect and involved burden and quality of life associated with COVID-19 (outpatient) | RCT | Emission factor from Ecoinvent, frame work from Griffiths et al, retrospective | 91 | 0.07 |  |
|  |  | 18 | INTERACT3 trial, University of Warwick | 7067 | 10 | 122 | 5.5 | High- cancer screening and involved mammographic surveillance for women after diagnosis of breast cancer | RCT | Emission factor from Ecoinvent, frame work, retrospective and prospective | 765 | 0.11 |  |
|  |  | 19 | INTERVAL, University of Cambridge | 2372 | 4 | 51 | 5.5 | Low- varying frequency of whole blood donation | Pragmatic trial | Emission factor from Ecoinvent, frame work, retrospective | 61 | 0.02 |  |
|  |  | 20 | MAVMET trial, University College, London | 90 | 1  (UK) | 6 | 5 | Low- drug for hepatic steatosis in HIV-1-infected adults (outpatient) | RCT | Emission factor from Ecoinvent, frame work, retrospective and prospective | 18 | 0.2 |  |
|  |  | 21 | ON-PACE trial, Imperial College London | 102 | 1  (UK) | 1 | 2.5 | Low- nutritional (supplement) effect and involved physical activity in patients with chronic obstructive pulmonary disease (COPD) | RCT | Emission factor from Ecoinvent, frame work, retrospective | 16 | 0.16 |  |
|  |  | 22 | PREMISE trial, Freeman Hospital | 536 | 3 | 10 | 5 | Medium- minimally invasive surgery- involved patients with bladder outlet obstruction due to enlarged prostate | RCT | Emission factor from Ecoinvent, frame work, retrospective | 25 | 0.05 |  |
|  |  | 23 | ReSTART trial, University Hospital, Lille | 537 | 4 | 112 | 8 | High- drug effect involved antiplatelet therapy after intracerebral hemorrhage (survivors, outpatient, include baseline MRI ) | RCT | Emission factor from Ecoinvent, frame work, retrospective | 109 | 0.20 |  |
|  |  | 24 | Shamrock trial, phase 2 Cancer Trials Ireland | 80 | 1 | 5 | 7 | Medium - drug effect and involved neoadjuvant treatment of patients with breast cancer (outpatient, include imaging) | RCT | Emission factor from Ecoinvent, frame work, retrospective and prospective | 58 | 0.72 |  |
|  |  | 25 | The UK stand together trial | 12,580 | 2 | 116 | 2,75 | Low - behavioral (complex intervention) - involved the effect of KiVa program in schools | Pragmatic trial | Emission factor from Ecoinvent, frame work, retrospective | 107 | 0.01 |  |
| **6** | **Griffiths et al.** (2024), by Institute of Cancer Research, London, UK and on behalf of the Low Carbon  Clinical Trials Group | 26 | CASPS, phase-2 trial, Institute of Cancer Research, UK | 47 | 3 | 12 | 8.50 | Medium - drug for patients with alveolar soft part sarcoma (CT scans, blood tests, outpatient-based ) | RCT | Emission factor from Ecoinvent, frame work, retrospective | 72 | 1.53 | H |
|  |  | 27 | PRIMETIME study, Cancer Research UK | 1962 | 4 | 64 | 14.8 | High - radiotherapy involved treatment for women with breast cancer | Pragmatic, trial | Emission factor from Ecoinvent, frame work, retrospective | 89 | 0.04 |  |
| **7** | **Fougerou-Leurent et al.** (2024), by Pharmacology Department, Rennes University Hospital, France | 28 | SUCRE, phase 3 trial, Rennes University Hospital, France | 202 | 1 (France) | 17 | 3.7 | High - treatment of chronic  subdural hematoma by corticosteroids | RCT | LCA- retrospective | 31.6 | 0.16 | H |
| **8** | **Mackillop et al.** (2023), by  AstraZeneca PLC,  Cambridge, UK | 29 | DAPA-HF trial, AstraZeneca | 4744 | 20 | 456 | 2.42 | Medium- drug effect in patients with chronic heart failure (echocardiography, outpatient) | RCT | LCA, Retrospective | 2498 | 0.53 | M |
|  |  | 30 | ADRIATIC, phase-3 trial, AstraZeneca | 730 | 19 | 261 | 7.42 | Medium - drug for patients with small-cell lung cancer (chemotherapy, outpatients) | RCT | LCA, Retrospective | 1638 | 2.24 |  |
|  |  | 31 | NAVIGATOR, phase-3 trial, AstraZeneca | 2000 | 29 | 300 | 2.75 | Medium - drug for adults and adolescents with severe uncontrolled asthma (outpatient) | RCT | LCA, Retrospective | 1437 | 0.71 |  |
| **9** | **Subaiya et al.** (2011), by Public Health and Environment, London School of Hygiene and Tropical  Medicine, UK | 32 | CRASH-1 trial, London School of Hygiene & Tropical Medicine | 10008 | 49 | 239 | 5 | High- drug for adults with head injury and some impairment of consciousness | RCT | One-year, activity-based audit; followed WBCSD reporting protocol; carbon calculated using mixed-source conversion factors, retrospective | 925 | 0.09 | M |
|  |  | 33 | CRASH-2 trial, London School of Hygiene & Tropical Medicine | 20211 | 40 | 247 | 5 | High- drug for bleeding trauma patients | RCT | One-year, activity-based audit; followed WBCSD reporting protocol; carbon calculated using mixed-source conversion factors, retrospective | 508 | 0.02 |  |
| **10** | **Lyle et al.*** (2009), by NIHR Evaluation, Trials and Studies Coordinating Centre, University of Southampton, UK | 34 | N/A, NIHR Health Technology Assessment funded trials | 201-300 | 1  (UK) | NA | 4.7 | Low- health technology assessment | Pragmatic trial | Activity-based audit; carbon calculated using standard conversion factors, retrospective | 42.1 | 0.18 | M |
|  |  | 35 | N/A, NIHR Health Technology Assessment funded trials | >500 | 1  (UK) | NA | 3.5 | Low- health technology assessment | Pragmatic trial | Activity-based audit; carbon calculated using standard conversion factors, retrospective | 46.7 | 0.08 |  |
|  |  | 36 | N/A, NIHR Health Technology Assessment funded trials | >500 | 1  (UK) | NA | 4.2 | Low- health technology assessment | Pragmatic trial | Activity-based audit; carbon calculated using standard conversion factors, retrospective | 52.6 | 0.09 |  |
|  |  | 37 | N/A, NIHR Health Technology Assessment funded trials | >500 | 1  (UK) | NA | 3.7 | Low- health technology assessment | Pragmatic trial | Activity-based audit; carbon calculated using standard conversion factors, retrospective | 72.0 | 0.13 |  |
|  |  | 38 | N/A, NIHR Health Technology Assessment funded trials | 301-500 | 1  (UK) | NA | 5.2 | Low- health technology assessment | Pragmatic trial | Activity-based audit; carbon calculated using standard conversion factors, retrospective | 72.9 | 0.21 |  |
|  |  | 39 | N/A, NIHR Health Technology Assessment funded trials | 0-200 | 1  (UK) | NA | 5.4 | Low- health technology assessment | Pragmatic trial | Activity-based audit; carbon calculated using standard conversion factors, retrospective | 76.0 | 0.88 |  |
|  |  | 40 | N/A, NIHR Health Technology Assessment funded trials | 201-300 | 1  (UK) | NA | 4.4 | Low- health technology assessment | Pragmatic trial | Activity-based audit; carbon calculated using standard conversion factors, retrospective | 85.3 | 0.39 |  |
|  |  | 41 | N/A, NIHR Health Technology Assessment funded trials | 201-300 | 1  (UK) | NA | 4.5 | Low- health technology assessment | Pragmatic trial | Activity-based audit; carbon calculated using standard conversion factors, retrospective | 86.2 | 0.40 |  |
|  |  | 42 | N/A, NIHR Health Technology Assessment funded trials | 301-500 | 1  (UK) | NA | 4.5 | Low- health technology assessment | Pragmatic trial | Activity-based audit; carbon calculated using standard conversion factors, retrospective | 91.2 | 0.20 |  |
|  |  | 43 | N/A, NIHR Health Technology Assessment funded trials | >500 | 1  (UK) | NA | 4.9 | Low- health technology assessment | Pragmatic trial | Activity-based audit; carbon calculated using standard conversion factors, retrospective | 101.5 | 0.09 |  |
|  |  | 44 | N/A, NIHR Health Technology Assessment funded trials | 301-500 | 1  (UK) | NA | 4.1 | Low- health technology assessment | Pragmatic trial | Activity-based audit; carbon calculated using standard conversion factors, retrospective | 102.0 | 0.32 |  |
|  |  | 45 | N/A, NIHR Health Technology Assessment funded trials | 0-200 | 1  (UK) | NA | 3.7 | Low- health technology assessment | Pragmatic trial | Activity-based audit; carbon calculated using standard conversion factors, retrospective | 112.7 | 0.70 |  |

Data were extracted from the included carbon footprint publications; when not reported in the included study, information was retrieved from external sources, including clinical study publications and clinical trial registries. Study duration is reported in years. For Lyle et al. (2009), total study duration was not reported; duration was estimated as total CO₂-eq divided by annual CO₂-eq (total CO₂ ÷ CO₂ per year). Total CO₂-eq is reported in tonnes. Per-participant CO₂-eq was calculated as total CO₂-eq divided by sample size, unless otherwise specified. Hospital resource demand was classified as low, medium, or high based on the intensity of trial-related procedures and infrastructure requirements, including clinical setting and use of diagnostic or therapeutic technologies.

Carbon footprint assessment approach refers to the method used to quantify CO₂-eq (life-cycle assessment, emission-factor-based methods, or activity-based audits) and the data collection direction (real time, retrospective, prospective, or mixed). Quality appraisal results are reported as high (H), medium (M), or low (L).

**Abbreviations:** CO₂-eq, carbon dioxide equivalent; RCT, randomized controlled trial; LCA, life-cycle assessment; QAR, quality appraisal result; RSV, respiratory syncytial virus; CT, computed tomography; MRI, magnetic resonance imaging; HIV, human immunodeficiency virus; COPD, chronic obstructive pulmonary disease; NIHR, National Institute for Health and Care Research; WBCSD, World Business Council for Sustainable Development

**Table S-3:** Inclusion and contribution of drug-manufacturing emissions to carbon-footprint estimates across primary clinical studies

| **Carbon-footprint assessment** | **Primary clinical study No.** | **Sample size** | **Estimated total CO₂-eq (t)** | **Drug-manufacturing emissions: inclusion and estimate (t CO₂e)** |
| --- | --- | --- | --- | --- |
| **LaRoche et al. (2025), 7 primary studies** | 1 | 39 | 17.6 | 0.9 |
|  | 2 | 255 | 580 | 255 |
|  | 3 | 125 | 1,146 | 597.3 |
|  | 4 | 517 | 3,107 | 1,715.4 |
|  | 5 | 178 | 359 | 11.7 |
|  | 6 | 1,124 | 208 | 64.5 |
|  | 7 | 276 | 171 | 72 |
| **Smit et al. (2025), 1 primary study** | 8 | 80 | 20.5 | 0.1* |
| **Cranley et al. (2025), 6 primary studies** | 9 | 300 | 26.3 | Excluded† |
|  | 10 | 54 | 8.8 | Excluded† |
|  | 11 | 194 | 47.6 | Excluded† |
|  | 12 | 363 | 54.9 | Excluded† |
|  | 13 | 800 | 19.1 | Excluded† |
|  | 14 | 537 | 106.4 | Excluded† |
| **Siegfried et al. (2025), 1 primary study** | 15 | 1,340 | 37.8 | Not applicable‡ |
| **Griffiths et al. (2024), 10 primary studies** | 16 | 12,580 | 74 | Excluded† |
|  | 17 | 537 | 91 | Excluded† |
|  | 18 | 102 | 765 | Excluded† |
|  | 19 | 1,245 | 61 | Excluded† |
|  | 20 | 90 | 18 | Excluded† |
|  | 21 | 536 | 16 | Excluded† |
|  | 22 | 7,067 | 25 | Excluded† |
|  | 23 | 535 | 109 | Excluded† |
|  | 24 | 11 | 58 | Excluded† |
|  | 25 | 2,372 | 107 | Excluded† |
| **Griffiths et al. (2024), 2 primary studies** | 26 | 48 | 72 | Excluded† |
|  | 27 | 1,962 | 89 | Not applicable‡ |
| **Fougerou-Leurent et al. (2024), 1 primary study** | 28 | 202 | 31.6 | Included; NR§ |
| **Mackillop et al. (2023), 3 primary studies** | 29 | 4,744 | 2,498 | 391.5¶ |
|  | 30 | 668 | 1,638 | 33¶ |
|  | 31 | 2,000 | 1,437 | 24¶ |
| **Subaiya et al. (2011), 2 primary studies** | 32 | 10,008 | 925 | Excluded† |
|  | 33 | 20,211 | 508 | Excluded† |
| **Lyle et al. (2009), 12 primary studies** | 34 | 240 | 4.73 | Included where applicable; NR# |
|  | 35 | 584 | 3.49 | Included where applicable; NR# |
|  | 36 | 585 | 4.17 | Included where applicable; NR# |
|  | 37 | 551 | 3.67 | Included where applicable; NR# |
|  | 38 | 344 | 5.24 | Included where applicable; NR# |
|  | 39 | 91 | 5.35 | Included where applicable; NR# |
|  | 40 | 220 | 4.42 | Included where applicable; NR# |
|  | 41 | 217 | 4.49 | Included where applicable; NR# |
|  | 42 | 453 | 4.49 | Included where applicable; NR# |
|  | 43 | 1,068 | 4.90 | Included where applicable; NR# |
|  | 44 | 317 | 4.08 | Included where applicable; NR# |
|  | 45 | 161 | 3.74 | Included where applicable; NR# |

**Abbreviation:** NR, not separately reported.

* Medication-manufacturing emissions were estimated using vancomycin and tenofovir as proxies for the antimalarial medications.
† Drug/IMP manufacturing was outside the scope of the carbon-footprint assessment.
‡ Drug manufacturing was not applicable because the underlying clinical study did not involve a pharmaceutical intervention.
§ Drug and placebo production and distribution were included, but associated emissions were not separately reported.
¶ Reported study-drug impact included manufacture, packaging and distribution of investigational product and placebo, administration where relevant, and waste treatment; manufacturing was not reported separately.
\# Pharmaceutical manufacture and delivery were included where applicable, but associated emissions were not separately reported.

**Table S-4:** Study-level carbon-footprint estimates with and without drug-production-related emissions in pharmaceutical trials

| **Assessment** | **Primary study No.** | **Total CO₂-eq (t)** | **Drug-production/study-drug emissions (t CO₂e)** | **Total minus drug-production/study-drug emissions (t CO₂e)** |
| --- | --- | --- | --- | --- |
| LaRoche et al. (2025) | 1 | 17.6 | 0.9 | 16.7 |
|  | 2 | 580 | 255 | 325.0 |
|  | 3 | 1,146 | 597.3 | 548.7 |
|  | 4 | 3,107 | 1,715.4 | 1,391.6 |
|  | 5 | 359 | 11.7 | 347.3 |
|  | 6 | 208 | 64.5 | 143.5 |
|  | 7 | 171 | 72 | 99.0 |
| Smit et al. (2025) | 8 | 20.5 | 0.1 | 20.4 |
| Mackillop et al. (2023) | 29 | 2,498 | 391.5 | 2,106.5 |
|  | 30 | 1,638 | 33 | 1,605.0 |
|  | 31 | 1,437 | 24 | 1,413.0 |
| **Total** | **Mean (SD)** | 1,016.6 (1,054.8) | 287.8 (511.7) | 728.8 (752.5) |
|  | **Median (IQR)** | 580.0 (189.5-1,537.5) | 64.5 (17.9-323.3) | 347.3 (121.3-1,402.3) |

The sensitivity analysis was restricted to pharmaceutical trials for which drug-production-related emissions were included in the assessment scope and separately quantifiable. Drug-production-related emissions were subtracted from the reported total CO₂-eq estimate, and summary statistics were recalculated. The total CO₂-eq column represents the originally reported estimates including drug-production-related emissions, whereas the final column represents the corresponding estimates after their exclusion.

**Table S-5:** Assessment of multicollinearity in the multivariable regression model

| **Predictor** | **Adjusted GVIF** |
| --- | --- |
| Duration | 1.36 |
| Sample size | 1.87 |
| Number of countries | 2.25 |
| Number of sites | 1.74 |
| Hospital resource demand | 1.28 |
| Carbon calculation system | 1.60 |

GVIF, generalized variance inflation factor. Adjusted GVIF refers to GVIF^(1/[2×df]), which accounts for the degrees of freedom (df) of predictors with multiple parameters and facilitates comparison across predictors. Higher values indicate greater multicollinearity [4]. All adjusted GVIF values were below commonly used thresholds for problematic multicollinearity, indicating no evidence of substantial multicollinearity among the predictors included in the model.

**Table S-6:** Identification of emission hotspots: distribution of CO₂-eq emissions across ten predefined activity groups in 45 clinical studies

| No. | **Authors** (year), number of included primary studies | Primary Study N# | Sample size | Estimated total  CO₂-eq (tonnes) | Hospital resource demand | CO2 calculation system | Emission activity category (tonnes CO₂-eq) | | | | | | | | | |
| --- | --- | --- | --- | --- | --- | --- | --- | --- | --- | --- | --- | --- | --- | --- | --- | --- |
|  |  |  |  |  |  |  | Trial setup | Clinical trial units | Meetings & travel | Intervention | Data collection | Trial supplies | Patient assessments | Samples | Laboratory | Trial close out |
| **1** | **LaRoche et al.** (2025),  Seven primary studies | 1 | 39 | 18 | Low | LCA | 3.8 | 4 | 5 | 0.8 | N/A | N/A | N/A | N/A | 0.8 | N/A |
|  |  | 2 | 255 | 580 | Medium | LCA | 104 | 45 | 53 | 255 | N/A | N/A | N/A | N/A | 121 | N/A |
|  |  | 3 | 125 | 1146 | Medium | LCA | 314 | 73 | 99 | 597 | N/A | N/A | N/A | N/A | 61 | N/A |
|  |  | 4 | 517 | 3107 | High | LCA | 473 | 87 | 480 | 1715 | N/A | N/A | N/A | N/A | 350 | N/A |
|  |  | 5 | 178 | 359 | Medium | LCA | 77 | 31 | 53 | 12 | N/A | N/A | N/A | N/A | 184 | N/A |
|  |  | 6 | 1124 | 208 | Medium | LCA | 95 | 11 | 36 | 64 | N/A | N/A | N/A | N/A | 0.1 | N/A |
|  |  | 7 | 276 | 171 | Medium | LCA | 39 | 15 | 42 | 71 | N/A | N/A | N/A | N/A | 0.4 | N/A |
| **2** | **Smit et al.** (2025)  One primary study | 8 | 80 | 20.5 | Low | LCA | 0.0 | 5.7 | 11.4 | 0.1 | 0.2 | 2.9 | 0.0 | 0.04 | 0.1 | 0.1 |
| **3** | **Cranley et al.** (2025)  Six primary studies | 9 | 300 | 26.3 | Low | non-LCA | 0.4 | 11.78 | 0.7 | 0.0 | 0.4 | 0.3 | 9.9 | 0.0 | 0.0 | 2.8 |
|  |  | 10 | 54 | 8.8 | High | non-LCA | 0.05 | 6.2 | 0.2 | 0.02 | 0.2 | 0.0 | 1.1 | 0.2 | 0.3 | 0.6 |
|  |  | 11 | 194 | 47.6 | Low | non-LCA | 0.1 | 23.2 | 0.5 | 0.1 | 1.1 | 7.4 | 14.2 | 0.0 | 0.0 | 1.2 |
|  |  | 12 | 363 | 54.9 | Low | non-LCA | 0.8 | 32.2 | 0.1 | 0.0 | 0.6 | 0.8 | 20.2 | 0.0 | 0.0 | 0.2 |
|  |  | 13 | 800 | 19.1 | High | non-LCA | 0.0 | 15.1 | 0.2\ | 0.3 | 2.01 | 1.55 | 0 | 0 | 0 | 19.1 |
|  |  | 14 | 537 | 106.4 | Medium | non-LCA | 1.09 | 78.87 | 4.25 | 0.0 | 2.2 | 6.4 | 13.1 | 0 | 0 | 0.5 |
| **4** | **Siegfried et al.** (2025)  One primary study | 15 | 1,340 | 37.8 | Low | non-LCA | 0.0 | 12.5 | 23.0 | 0.0 | 0.0 | 2.3 | 0 | 0 | 0 | 0 |
| **5** | **Griffiths et al.** (2024), Ten primary studies | 16 | 12580 | 74 | Medium- | non-LCA | 0.5 | 37.2 | 14.9 | 0.02 | 0.2 | 52.9 | N/A | N/A | N/A | 1.4 |
|  |  | 17 | 537 | 91 | Low | non-LCA | 1.4 | 78.9 | 4.3 | 2.1 | 2.1 | 6.4 | 11.4 | N/A | N/A | 4.2 |
|  |  | 18 | 102 | 765 | High | non-LCA | 0.04 | 5 | 0.02 | 0.4 | 0.03 | 0.6 | 2.6 | 0.05 | 5.7 | 1.2 |
|  |  | 19 | 1245 | 61 | Low- | non-LCA | 0.2 | 15.6 | 0.1 | N/A | 72.1 | 0.1 | N/A | N/A | N/A | 2.5 |
|  |  | 20 | 90 | 18 | Low | non-LCA | 0.02 | 2.1 | 0.9 | 0.3 | 0.2 | 0.9 | 7.1 | 0.06 | 2.7 | 3.6 |
|  |  | 21 | 536 | 16 | Low | non-LCA | 0.7 | 13.5 | 2.7 | N/A | 0.3 | 0.4 | 6.8 | N/A | N/A | 0.6 |
|  |  | 22 | 7067 | 25 | Medium | non-LCA | 13.7 | 546.2 | 49.9 | 48.1 | 3.3 | N/A | 62 | N/A | N/A | 41.3 |
|  |  | 23 | 535 | 109 | High | non-LCA | 0.05 | 19.6 | 11.1 | 0.1 | 0.1 | 12.6 | 18.2 | 0.005 | 3.6 | 9 |
|  |  | 24 | 11 | 58 | Medium | non-LCA | 0.2 | 6.8 | 4.6 | 0.7 | 0.1 | 23.7 | 1.3 | N/A | 17.8 | 2.5 |
|  |  | 25 | 2372 | 107 | Low | non-LCA | 1 | 10.9 | 27.8 | N/A | 0.7 | 5.3 | 13.1 | N/A | N/A | 2.2 |
| **6** | **Griffiths et al.** (2024),  Two primary studies | 26 | 48 | 72 | Medium | non-LCA | 0.4 | 23.5 | 20.5 | 1.3 | 0.6 | 0.5 | 11.3 | 1.9 | 9.4 | 2.7 |
|  |  | 27 | 1962 | 89 | High | non-LCA | 0.5 | 11.7 | 1.1 | N/A | 7.6 | 1.7 | 60.8 | 0.4 | 2.1 | 3.4 |
| **7** | **Fougerou-Leurent et al.** (2024),  One primary study | 28 | 202 | 31.6 | High | LCA | N/A | N/A | N/A | N/A | N/A | N/A | N/A | N/A | N/A | N/A |
| **8** | **Mackillop et al.** (2023),  Three primary studies | 29 | 4744 | 2498 | Medium | LCA | 113.9 | 361.4 | 1017.9 | N/A | 70.9 | 37.6 | 144.5 | 483 | 268.9 | N/A |
|  |  | 30 | 668 | 1638 | Medium | LCA | 127.9 | 838 | 387 | N/A | 95 | 87.3 | 11 | 47.1 | 45.1 | N/A |
|  |  | 31 | 2000 | 1437 | Medium | LCA | 225.4 | 19 | 280.8 | N/A | 54 | 503.3 | 128 | 45.4 | 181 | N/A |
| **9** | **Subaiya et al.** (2011),  Two primary studies | 32 | 10008 | 925 | High | non-LCA | N/A | 278.46 | 17.34 | N/A | N/A | N/A | 190.23 | 438.6 | N/A | N/A |
|  |  | 33 | 20211 | 508 | High | non-LCA | N/A | 185.65 | 15.98 | N/A | N/A | N/A | 147.11 | 160.74 | N/A | N/A |
| **10** | **Lyle et al.** (2009),  Twelve primary studies | 34 | 240 | 4.73 | Low | non-LCA | N/A | 18.1 | 35.6 | N/A | N/A | 12.2 | 12.6 | N/A | N/A | N/A |
|  |  | 35 | 584 | 3.49 | Low | non-LCA | N/A | 18.1 | 35.6 | N/A | N/A | N/A | 12.6 | N/A | N/A | N/A |
|  |  | 36 | 585 | 4.17 | Low | non-LCA | N/A | 18.1 | 35.6 | N/A | N/A | N/A | 12.6 | N/A | N/A | N/A |
|  |  | 37 | 551 | 3.67 | Low | non-LCA | N/A | 18.1 | 35.6 | N/A | N/A | N/A | 12.6 | N/A | N/A | N/A |
|  |  | 38 | 344 | 5.24 | Low | non-LCA | N/A | 18.1 | 35.6 | N/A | N/A | N/A | 12.6 | N/A | N/A | N/A |
|  |  | 39 | 91 | 5.35 | Low | non-LCA | N/A | 18.1 | 35.6 | N/A | N/A | N/A | 12.6 | N/A | N/A | N/A |
|  |  | 40 | 220 | 4.42 | Low | non-LCA | N/A | 18.1 | 35.6 | N/A | N/A | N/A | 12.6 | N/A | N/A | N/A |
|  |  | 41 | 217 | 4.49 | Low | non-LCA | N/A | 18.1 | 35.6 | N/A | N/A | N/A | 12.6 | N/A | N/A | N/A |
|  |  | 42 | 453 | 4.49 | Low | non-LCA | N/A | 18.1 | 35.6 | N/A | N/A | N/A | 12.6 | N/A | N/A | N/A |
|  |  | 43 | 1,068 | 4.90 | Low | non-LCA | N/A | 18.1 | 35.6 | N/A | N/A | N/A | 12.6 | N/A | N/A | N/A |
|  |  | 44 | 317 | 4.08 | Low | non-LCA | N/A | 18.1 | 35.6 | N/A | N/A | N/A | 12.6 | N/A | N/A | N/A |
|  |  | 45 | 161 | 3.74 | Low | non-LCA | N/A | 18.1 | 35.6 | N/A | N/A | N/A | 12.6 | N/A | N/A | N/A |
| **Number of times each domain was the highest emitter** | | | | | | | **1** | **13** | **17** | **4** | **1** | **3** | **3** | **1** | **2** | **0** |

The table summarizes estimated carbon dioxide emissions (tCO₂e) across ten predefined groups of activities for 45 clinical studies. Activities groups follow the classification proposed by Griffiths et al. (2024) focusing on study-related activities beyond routine care. For each study, the activity contributing the highest emissions was identified as *an emission hotspot* and is highlighted using **thicker borders**. The total number of times each domain was the highest emitter has been specified in the last row.

If study activities were reported using a different categorization, we harmonized the data by reassigning each activity to the most appropriate Griffiths et al. domain (based on the description of the activity) and all estimates expressed in tCO₂e for comparability. When activity-specific emissions were not reported, the corresponding activities were coded as N/A, indicating unavailable data rather than confirmed absence of emissions.

Each **activity category** captures the following activities:

- **Trial setup:** Production and provision of documentation to sites or patients.
- **Clinical Trial Unit (CTU) emissions:** Energy consumption of trial staff working in an office and commuting or working from home for the duration of the trial.
- **Trial-specific meetings and travel:** Teleconferencing, trial staff travel, sustenance and hotel stays for meetings, site visits, audits and conferences.
- **Treatment (intervention):** Shipment of intervention from manufacturer to distributor and/or sites/participants, packaging of intervention and destruction of overage. Manufacture of IMP or other intervention is excluded.
- **Data collection and exchange:** Data collection and storage, for example, emails, trial databases, data linkage, questionnaires, Case Report Forms (CRFs).
- **Trial supplies and equipment:** Equipment used by CTU, supplied to sites or to participants specifically for the trial, for example, IT equipment and wearables, laboratory equipment.
- **Trial-specific patient assessments:** Patient travel and hospital staff time required for trial-specific assessments, for example, scans, bloods, bed days.
- Only activities undertaken to answer the research question that are in addition to routine care are included.
- **Samples**: Sample kit manufacture and shipment from CTU to sites.
- **Laboratory:** Sample analysis/processing, storage at a central laboratory and/or site laboratories.
- **Trial close out:**  Archiving of documentation and ambient samples, return of supplies

**Table S-7:** PRISMA reporting checklist for the systematic review

| **Section and Topic** | **Item #** | **Checklist item** | **Location where item is reported (page number)** |
| --- | --- | --- | --- |
| **TITLE** | | |  |
| Title | 1 | Identify the report as a systematic review. | 1 |
| **ABSTRACT** | | |  |
| Abstract | 2 | See the PRISMA 2020 for Abstracts checklist. | - |
| **INTRODUCTION** | | |  |
| Rationale | 3 | Describe the rationale for the review in the context of existing knowledge. | 4 |
| Objectives | 4 | Provide an explicit statement of the objective(s) or question(s) the review addresses. | 4-5 |
| **METHODS** | | |  |
| Eligibility criteria | 5 | Specify the inclusion and exclusion criteria for the review and how studies were grouped for the syntheses. | 6  Methods S-1 |
| Information sources | 6 | Specify all databases, registers, websites, organisations, reference lists and other sources searched or consulted to identify studies. Specify the date when each source was last searched or consulted. | 6  Methods S-2 |
| Search strategy | 7 | Present the full search strategies for all databases, registers and websites, including any filters and limits used. | Methods S-2 |
| Selection process | 8 | Specify the methods used to decide whether a study met the inclusion criteria of the review, including how many reviewers screened each record and each report retrieved, whether they worked independently, and if applicable, details of automation tools used in the process. | 6-7  Figure S-1 |
| Data collection process | 9 | Specify the methods used to collect data from reports, including how many reviewers collected data from each report, whether they worked independently, any processes for obtaining or confirming data from study investigators, and if applicable, details of automation tools used in the process. | 6-7 |
| Data items | 10a | List and define all outcomes for which data were sought. Specify whether all results that were compatible with each outcome domain in each study were sought (e.g. for all measures, time points, analyses), and if not, the methods used to decide which results to collect. | 6-7 |
|  | 10b | List and define all other variables for which data were sought (e.g. participant and intervention characteristics, funding sources). Describe any assumptions made about any missing or unclear information. | 6-7 |
| Study risk of bias assessment | 11 | Specify the methods used to assess risk of bias in the included studies, including details of the tool(s) used, how many reviewers assessed each study and whether they worked independently, and if applicable, details of automation tools used in the process. | 7-8  Methods S-4 |
| Effect measures | 12 | Specify for each outcome the effect measure(s) (e.g. risk ratio, mean difference) used in the synthesis or presentation of results. | 8 |
| Synthesis methods | 13a | Describe the processes used to decide which studies were eligible for each synthesis (e.g. tabulating the study intervention characteristics and comparing against the planned groups for each synthesis (item #5)). | 8 |
|  | 13b | Describe any methods required to prepare the data for presentation or synthesis, such as handling of missing summary statistics, or data conversions. | 8-9 |
|  | 13c | Describe any methods used to tabulate or visually display results of individual studies and syntheses. | 6-7 |
|  | 13d | Describe any methods used to synthesize results and provide a rationale for the choice(s). If meta-analysis was performed, describe the model(s), method(s) to identify the presence and extent of statistical heterogeneity, and software package(s) used. | 8-9 |
|  | 13e | Describe any methods used to explore possible causes of heterogeneity among study results (e.g. subgroup analysis, meta-regression). | 8-9 |
|  | 13f | Describe any sensitivity analyses conducted to assess robustness of the synthesized results. | 8-9  Weighted and unweighted synthesis |
| Reporting bias assessment | 14 | Describe any methods used to assess risk of bias due to missing results in a synthesis (arising from reporting biases). | - |
| Certainty assessment | 15 | Describe any methods used to assess certainty (or confidence) in the body of evidence for an outcome. | - |
| **RESULTS** | | |  |
| Study selection | 16a | Describe the results of the search and selection process, from the number of records identified in the search to the number of studies included in the review, ideally using a flow diagram. | Figure S-1 |
|  | 16b | Cite studies that might appear to meet the inclusion criteria, but which were excluded, and explain why they were excluded. | Table S-4 |
| Study characteristics | 17 | Cite each included study and present its characteristics. | 10 |
| Risk of bias in studies | 18 | Present assessments of risk of bias for each included study. | Table 2 |
| Results of individual studies | 19 | For all outcomes, present, for each study: (a) summary statistics for each group (where appropriate) and (b) an effect estimate and its precision (e.g. confidence/credible interval), ideally using structured tables or plots. | Table 1 |
| Results of syntheses | 20a | For each synthesis, briefly summarise the characteristics and risk of bias among contributing studies. | 10-11 |
|  | 20b | Present results of all statistical syntheses conducted. If meta-analysis was done, present for each the summary estimate and its precision (e.g. confidence/credible interval) and measures of statistical heterogeneity. If comparing groups, describe the direction of the effect. | 11-12 |
|  | 20c | Present results of all investigations of possible causes of heterogeneity among study results. | 12 |
|  | 20d | Present results of all sensitivity analyses conducted to assess the robustness of the synthesized results. | 12 |
| Reporting biases | 21 | Present assessments of risk of bias due to missing results (arising from reporting biases) for each synthesis assessed. | - |
| Certainty of evidence | 22 | Present assessments of certainty (or confidence) in the body of evidence for each outcome assessed. | - |
| **DISCUSSION** | | |  |
| Discussion | 23a | Provide a general interpretation of the results in the context of other evidence. | 13 |
|  | 23b | Discuss any limitations of the evidence included in the review. | 16 |
|  | 23c | Discuss any limitations of the review processes used. | 16 |
|  | 23d | Discuss implications of the results for practice, policy, and future research. | 14-16 |
| **OTHER INFORMATION** | | |  |
| Registration and protocol | 24a | Provide registration information for the review, including register name and registration number, or state that the review was not registered. | 6 |
|  | 24b | Indicate where the review protocol can be accessed, or state that a protocol was not prepared. | 6 |
|  | 24c | Describe and explain any amendments to information provided at registration or in the protocol. | N/A |
| Support | 25 | Describe sources of financial or non-financial support for the review, and the role of the funders or sponsors in the review. | 18 |
| Competing interests | 26 | Declare any competing interests of review authors. | 18 |
| Availability of data, code and other materials | 27 | Report which of the following are publicly available and where they can be found: template data collection forms; data extracted from included studies; data used for all analyses; analytic code; any other materials used in the review. | 18 |

**Table S-8.** Potentially relevant studies excluded from this systematic review and reasons for exclusion

| **Number** | **Reference** | **Reason for exclusion** |
| --- | --- | --- |
| 1 | Sustainable Trials Study Group. Towards sustainable clinical trials. *BMJ*. 2007 Mar 29;334(7595):671–3. | Repeated trial already included in another study |
| 2 | LaRoche JK, Alvarenga R, Collins M, Costelloe T, De Soete W, Faludi J, Rens K. Climate footprint of industry-sponsored clinical research: an analysis of a phase-1 randomised clinical study and discussion of opportunities to reduce its impact. *BMJ Open*. 2024 Jan 1;14(1):e077129. | Repeated trial already included in another study |
| 3 | Adshead F, Salman RA, Aumonier S, Collins M, Hood K, McNamara C, Moore K, Smith R, Sydes MR, Williamson PR. A strategy to reduce the carbon footprint of clinical trials. *The Lancet*. 2021 Jul 24;398(10297):281–2. | Commentary; estimate based on multiplying the number of trials by the average carbon footprint per trial from the CRASH-1 study |
| 4 | Pencheon DC. Managing the environmental impact of research. *Trials*. 2011 Mar 16;12(1):80. | Refers to data reported by Subaiya et al. |
| 5 | Klasen J, Horstink MM, Hunfeld NG. Include environmental sustainability in clinical trial design and implementation strategies: the BLING-III trial as an illustrative example. *Intensive Care Medicine*. 2025 Feb;51(2):440–1. | They do not calculate a total CO₂e (tonnes) for conducting the BLING-III trial |
| 6 | Bayrau BA, Buyukcangaz E, Sadarangani SP, Ondigo BN, Prinzi A, LaBeaud AD. (Un) Sustainable Science: Greening Practices in Research, Clinical Microbiology, and Veterinary Laboratories Locally and Globally. In *Open Forum Infectious Diseases*. 2025 Feb (Vol. 12, No. 2, p. ofae677). US: Oxford University Press. | Not an original calculation performed in this OFID review |

**References:**

[1] ISO. ISO 14040. International Organization for Standardization: Switzerland: environmental management - life cycle assessment -requirements and guidelines; 1997.

[2] ISO. ISO 14044. International Organization for Standardization: Switzerland: environmental management - life cycle assessment -requirements and guidelines; 2006.

[3] Huijbregts, M.A.J., Steinmann, Z.J.N., Elshout, P.M.F., Stam, G., Verones, F., Vieira, M., et al., 2017. ReCiPe2016: a harmonised life cycle impact assessment method at midpoint and endpoint level. Int. J. Life Cycle Assess. 22 (2), 138–147.

[4] O’brien RM. A caution regarding rules of thumb for variance inflation factors. Quality & quantity. 2007 Oct;41(5):673-90.
